# Aerobic and Aerobic-Oriented Exercise for Anthropometric, Androgen-Related, and Reproductive Endocrine Outcomes in Polyendocrine Metabolic Ovarian Syndrome (Polycystic Ovary Syndrome): A Systematic Review and Meta-Analysis

**DOI:** 10.3390/metabo16100745

**Published:** 2026-10-03

**Authors:** Lanxin Qiu, Yunzhi Emma Huang, Mingnan Zhuang, Juntao Kan, Zhengqi Qiu

**Affiliations:** 1 Department of Applied Psychology, School of Health Management, Guangzhou Medical University, Guangzhou 511436, China; 2025026@tea.xaipe.edu.cn; 2 School of Physical Education and Training, Xi’an Physical Education University, No. 1 Dongcheng South Road, Huyi District, Xi’an 710399, China; 3 Nutrilite Health Institute, Amway R&D Center, 720 Cailun Road, Shanghai 201203, China; emma.huang@amway.com; 4 College of Sports Training, Tianjin University of Sport, Tianjin 301617, China; zmingnan027@stu.tjus.edu.cn

**Keywords:** polycystic ovary syndrome, polyendocrine metabolic ovarian syndrome, aerobic exercise, sex hormone-binding globulin, androgen, meta-analysis

## Abstract

**Background:** Aerobic exercise is recommended within lifestyle management for polycystic ovary syndrome (PCOS), but its effects on anthropometric and endocrine outcomes remain inconsistent. **Methods:** Five databases were searched from inception through July 2026. Controlled trials of structured aerobic or aerobic-oriented exercise lasting at least four weeks were eligible when the comparator did not receive an exercise program expected to improve aerobic fitness. Between-group changes were expressed as Hedges’ g and pooled using random-effects models with cluster-robust small-sample inference. Subgroup, univariable meta-regression, and dose–response analyses were exploratory. **Results:** Twenty-two reports representing 20 independent trials were included. The pooled standardized estimate marginally favored exercise for body weight (g = −0.09, 95% CI −0.13 to −0.05), although its magnitude was trivial and the 95% prediction interval crossed the null (−0.29 to 0.11). Exercise was associated with lower body mass index (g = −0.20, 95% CI −0.33 to −0.06), waist-to-hip ratio (g = −0.36, 95% CI −0.56 to −0.19), total testosterone (g = −0.43, 95% CI −0.67 to −0.20), and free testosterone (g = −0.60, 95% CI −0.99 to −0.21), and with higher sex hormone-binding globulin (SHBG; g = 0.24, 95% CI 0.04 to 0.44). No clear pooled effects were observed for waist circumference, body fat percentage, free androgen index, dehydroepiandrosterone sulfate, or anti-Müllerian hormone. Follicle-stimulating hormone and luteinizing hormone estimates lay at the statistical boundary and were heterogeneous; leave-one-study-out inference was also unstable for free testosterone and SHBG. Certainty was low or very low across outcomes. **Conclusions:** Aerobic or aerobic-oriented exercise showed favorable pooled differences in selected anthropometric and androgen-related markers, but low certainty and the sensitivity of some estimates preclude an endocrine-targeted dose or reproductive-benefit claim.

## 1. Introduction

Following a multistep global consensus process, polycystic ovary syndrome (PCOS) was renamed polyendocrine metabolic ovarian syndrome (PMOS) to better reflect its endocrine, metabolic, and ovarian manifestations [1]. The studies synthesized in this review recruited participants under established PCOS diagnostic criteria. We therefore use PCOS when describing the study populations and evidence, while acknowledging PMOS as the new name. No diagnostic reclassification was undertaken for this review. The renaming reflects a consensus on nomenclature and on how the condition is characterized; it was not tested by, and is not evaluated in, the trials synthesized here.

PCOS is heterogeneous: hyperandrogenism, ovulatory dysfunction, and adverse metabolic phenotypes occur in different combinations and with varying severity. Aerobic exercise is particularly relevant to this heterogeneous condition because it can enhance cardiorespiratory fitness, increase energy expenditure, support weight management and body composition, and contribute to better metabolic health, including reductions in central adiposity and improvements in lipid profile. Importantly, lifestyle-related health benefits may occur even in the absence of clinically meaningful weight loss [2]. Consistent with these benefits, international evidence-based guidance recommends lifestyle intervention and physical activity and includes moderate- or vigorous-intensity aerobic activity within general health targets. However, these established general and metabolic benefits should not be assumed to translate uniformly into improvements in androgen-related or reproductive endocrine outcomes. No exercise modality or intensity has established superiority for anthropometric, metabolic, hormonal, reproductive, or psychological outcomes. The outcome-specific endocrine effects of aerobic exercise therefore remain clinically relevant but incompletely defined.

Randomized trials have examined endocrine responses to aerobic training in women with PCOS [3], and previous meta-analyses have evaluated exercise across multiple modalities, reporting null overall androgen estimates with a positive SHBG signal confined to an aerobic subgroup [4]. Other reviews have addressed exercise and dietary co-intervention comparisons [5], focused on anti-Müllerian hormone (AMH) [6], or examined multicomponent yoga interventions [7]. The remaining uncertainty concerns the magnitude, consistency, and certainty of individual endocrine responses within a defined exercise–comparator contrast. An outcome-specific synthesis of the aerobic and aerobic-oriented interventions considered here can complement this evidence by evaluating total and free testosterone, the free androgen index (FAI), sex hormone-binding globulin (SHBG), and reproductive endocrine markers separately alongside anthropometric outcomes. This can clarify the pattern of reported biochemical responses and the uncertainty surrounding their interpretation. Eligibility here was further restricted to an aerobic or aerobic-oriented intervention contrasted against a comparator not expected to improve aerobic fitness, with co-interventions permitted only when delivered equally to both groups so that the pooled contrast is attributable to the exercise stimulus rather than to a combined lifestyle package.

This systematic review and meta-analysis therefore evaluated the effects of structured aerobic or aerobic-oriented exercise on anthropometric, androgen-related, and reproductive endocrine outcomes in women diagnosed with PCOS. Each outcome was synthesized separately using an analytical framework that accounted for dependent estimates from the same trial and incorporated prediction intervals, sensitivity analyses, and outcome-level certainty assessment to characterize effect magnitudes and their uncertainty. Study-level participant and exercise-prescription characteristics were also examined; all subgroup, meta-regression, and dose–response analyses were exploratory and hypothesis-generating.

## 2. Materials and Methods

### 2.1. Protocol and Reporting

This systematic review and meta-analysis followed a prespecified protocol and was reported in accordance with the Preferred Reporting Items for Systematic Reviews and Meta-Analyses (PRISMA) 2020 statement [8]. Risk of bias was assessed using the Cochrane Risk of Bias 2 tool [9]. The PRISMA for Abstracts and full-report checklists are provided in Supplementary Tables S1 and S2. The protocol was registered in the International Prospective Register of Systematic Reviews (PROSPERO; CRD420261475528).

### 2.2. Information Sources and Search Strategy

PubMed/MEDLINE, Embase, Web of Science Core Collection, the Cochrane Library, and Scopus were searched from database inception through July 2026. No restrictions were imposed on publication year or sample size. Database searches were supplemented by screening the reference lists of eligible studies, forward citation tracking, and the related-article functions in PubMed and Embase. The complete database-specific strategies are reported in Supplementary Tables S3–S7.

The population concept combined controlled vocabulary and free-text terms for both the current and the former name of the condition—polyendocrine metabolic ovarian syndrome and polycystic ovary syndrome—together with historical synonyms, including Stein–Leventhal syndrome, so that retrieval was not conditional on the nomenclature used by a given report. The intervention concept included exercise, exercise therapy, physical activity, aerobic and endurance training, walking, running, cycling, swimming, continuous training, and interval training. Outcome terms were not included in the search to avoid restricting retrieval.

### 2.3. Eligibility Criteria

Eligibility was defined according to the Population, Intervention, Comparator, Outcomes, and Study design framework. Participants were women diagnosed with PCOS according to criteria recognized when the study was conducted, including the National Institutes of Health, Rotterdam, or Androgen Excess and PCOS Society criteria. Because the renaming did not introduce new diagnostic criteria, eligibility was unaffected by the change in nomenclature. Eligible interventions comprised structured, repeated aerobic or aerobic-oriented exercise lasting at least four weeks. An intervention was classified as aerobic when the prescribed stimulus was continuous or interval dynamic activity engaging large muscle groups at an intensity within the aerobic training range defined by the American College of Sports Medicine [10]. An intervention was classified as aerobic-oriented when continuous, rhythmic whole-body movement was the principal prescribed content of each session, but the session was not delivered as conventional endurance training; yoga and Tai Chi were classified in this category on that basis. Interventions in which movement was not the principal prescribed content, and interventions whose principal prescribed stimulus was progressive external resistance, were not eligible. Because this category is heterogeneous, modality was retained as a prespecified subgroup variable, and mind–body interventions were analyzed as a separate modality category rather than pooled with conventional endurance training. Co-interventions were permitted only when delivered equally to the intervention and control groups. Eligible comparators included usual care, wait-list control, maintenance of habitual activity, no exercise, or a minimal intervention not intended to improve aerobic fitness. Parallel-group and eligible multiarm controlled trials with a concurrent comparator were included irrespective of whether allocation was randomized, quasi-randomized, or non-randomized; the allocation method was extracted for every report and carried into the risk-of-bias assessment. For multiarm trials, only contrasts between an eligible exercise arm and an eligible non-aerobic control were extracted.

Prespecified anthropometric outcomes were body weight, body mass index (BMI), waist and hip circumferences, waist-to-hip ratio, body fat percentage, fat mass, and lean or fat-free mass. Androgen-related outcomes were total testosterone, free testosterone, FAI, dehydroepiandrosterone sulfate (DHEAS), and SHBG. Reproductive endocrine outcomes were AMH, follicle-stimulating hormone (FSH), luteinizing hormone (LH), estradiol, progesterone, and prolactin. Hormonal outcomes were required to have been measured in serum or plasma. All androgen-related and reproductive endocrine outcomes were circulating biochemical markers and were prespecified as surrogate outcomes; patient-important reproductive outcomes, including menstrual regularity, ovulation, pregnancy, live birth, and hirsutism, were not synthesized.

Studies were excluded when they evaluated an acute exercise bout or an intervention lasting less than four weeks; combined aerobic-resistance, pharmacological, dietary, supplementation, or multicomponent lifestyle interventions from which the independent exercise effect could not be isolated; an active exercise comparator without an eligible non-aerobic control; or insufficient outcome information for effect estimation. Secondary reports supplying unique outcome data were linked to the parent trial; reports without original outcome data, peer-reviewed full text, or English-language text were excluded. Reports arising from the same or overlapping trial population were treated as a single trial to avoid double counting.

### 2.4. Study Selection and Data Extraction

Records were imported into EndNote X9, deduplicated electronically, and verified manually. Two reviewers independently screened titles and abstracts and then assessed the full texts of potentially eligible reports. Exclusion reasons were recorded at full-text review. Disagreements were resolved by discussion and, when required, by a third reviewer.

Two reviewers independently extracted study design, diagnostic criteria, participant characteristics, intervention and comparator content, randomized and analyzed denominators, exercise modality and prescription, supervision, follow-up, and outcome data. Baseline, post-intervention, or change values were extracted with their standard deviations, measurement units, and analyzed sample sizes. Discrepancies were resolved by consensus or third-reviewer adjudication. When numerical data were available only graphically and could not be obtained from the study authors, WebPlotDigitizer version 4.1 was used.

### 2.5. Risk of Bias

Risk of bias was assessed using the Cochrane Risk of Bias 2 tool [9]. The five domains addressed the randomization process, deviations from intended interventions, missing outcome data, outcome measurement, and selection of the reported result. Two reviewers independently assigned domain-level and overall judgments of low risk, some concerns, or high risk and resolved disagreements by consensus or third-reviewer adjudication. The same instrument was applied to every included trial so that judgments remained comparable across the evidence base; for trials without an explicitly randomized allocation sequence, this is reflected in the randomization-process domain.

### 2.6. Exercise-Prescription Coding

Exercise intensity and dose were coded using the frequency, intensity, time, type, volume, and progression framework and measure-specific thresholds from the American College of Sports Medicine [10]. Reported physiological measures, including percentages of maximal oxygen uptake, maximal heart rate, and heart-rate reserve, were classified using their corresponding thresholds rather than converted directly across measures. The prescribed dose was used because the achieved dose and adherence were not reported consistently.

Metabolic equivalent of task (MET) values reported by the original studies were preferred. When unavailable, the closest activity code in the 2024 Adult Compendium of Physical Activities was selected from the reported modality, speed, power, incline, or perceived exertion [11]. For mind–body modalities, MET values were taken from the corresponding Compendium activity codes rather than from endurance-exercise codes. Session duration represented the main aerobic component when warm-up and cool-down periods were reported separately. Midpoints were used for reported ranges, time-weighted means were calculated for progressive protocols, and interval work and recovery phases were integrated according to their respective duration and intensity. Dose metrics were expressed as weekly exercise time, MET-min per session, and MET-min per week.

### 2.7. Effect-Size Calculation and Quantitative Synthesis

The primary effect was the between-group difference in change from baseline. When change-score standard deviations were not reported, they were derived from baseline and follow-up variances using a prespecified within-group correlation of r = 0.50 [12]. Because most trials were small, standardized effects were expressed as Hedges’ g with the small-sample correction [13,14]. Negative values indicate a greater reduction with exercise, and positive values indicate a greater increase. Absolute magnitudes were described as trivial (<0.20), small (0.20–0.49), moderate (0.50–0.79), or large (≥0.80) [15].

Analyses were performed in R (version 4.4.3) using the metafor and clubSandwich packages [16,17]. Outcome-specific random-effects models were fitted with restricted maximum-likelihood estimation [18]. When a trial contributed more than one eligible intervention arm or related estimate, a sampling variance–covariance matrix was constructed using a working within-trial correlation of ρ = 0.60. The primary two-level multivariate model included a trial-level random intercept. Statistical inference used cluster-robust variance estimation with the CR2 small-sample adjustment and Satterthwaite degrees of freedom, clustered by independent trial, as implemented in clubSandwich [17,19]. Pooled estimates are reported with 95% confidence intervals (CIs) and two-sided *p* values; *p* < 0.05 was the prespecified statistical threshold.

Heterogeneity was summarized using Cochran’s Q, I^2^, tau2, and tau [20,21]. I^2^ values of 0–25%, >25–50%, >50–75%, and >75% were described as low, moderate, high, and very high heterogeneity, respectively. Ninety-five percent prediction intervals were calculated from the fitted random-effects models to describe the range expected for the true effect in a comparable new study [22,23].

### 2.8. Exploratory Moderator and Dose–Response Analyses

Categorical moderators were examined with subgroup analyses, and continuous moderators with separate univariable meta-regressions. Consistent with methodological guidance, these analyses were restricted to outcomes with at least 10 independent trials; formal subgroup-difference tests additionally required at least three trials per category [12,24]. Prespecified subgroup variables were baseline obesity status, exercise modality, exercise intensity, and supervision. Continuous moderators included mean age, baseline BMI, warm-up duration, intervention duration, session duration, and weekly exercise frequency.

Exploratory dose–response models were fitted for body mass index, total testosterone, and sex hormone-binding globulin, which were the outcomes for which at least 10 independent trials provided sufficient dose information. Separate random-effects models included linear and quadratic terms for MET-min per session, weekly exercise time, and MET-min per week. These analyses retained the covariance structure and CR2 inference used in the primary models. Because multiple study-level moderator models were examined without multiplicity adjustment and observations were sparse at some exposure levels, the results were interpreted as exploratory signals rather than confirmatory evidence.

### 2.9. Small-Study Effects and Sensitivity Analyses

Contour-enhanced funnel plots [25] and the Egger regression [26,27] were used to examine small-study effects when at least 10 independent trials were available [28]. Egger models retained the within-trial covariance structure and CR2 inference. Funnel asymmetry was interpreted as a potential small-study effect rather than direct evidence of publication bias.

Robustness was examined by comparing two- and three-level random-effects models, varying the working within-trial correlation from ρ = 0.40 to 0.80, and repeating the primary model after sequential omission of each independent study.

### 2.10. Certainty of Evidence

Outcome-level certainty was assessed using the Grading of Recommendations Assessment, Development, and Evaluation (GRADE) approach [29]. Randomized-trial evidence began at high certainty and could be rated down for risk of bias, inconsistency, indirectness, imprecision, or publication bias. One reviewer conducted the initial assessment, and a second reviewer verified each judgment. Because the review question concerned these prespecified biochemical and anthropometric outcomes themselves, indirectness was judged in relation to those outcomes rather than to patient-important reproductive outcomes; the resulting ratings therefore describe certainty about the surrogate markers and not about ovarian function, fertility, or symptom outcomes.

## 3. Results

### 3.1. Study Selection

**Figure 1:**
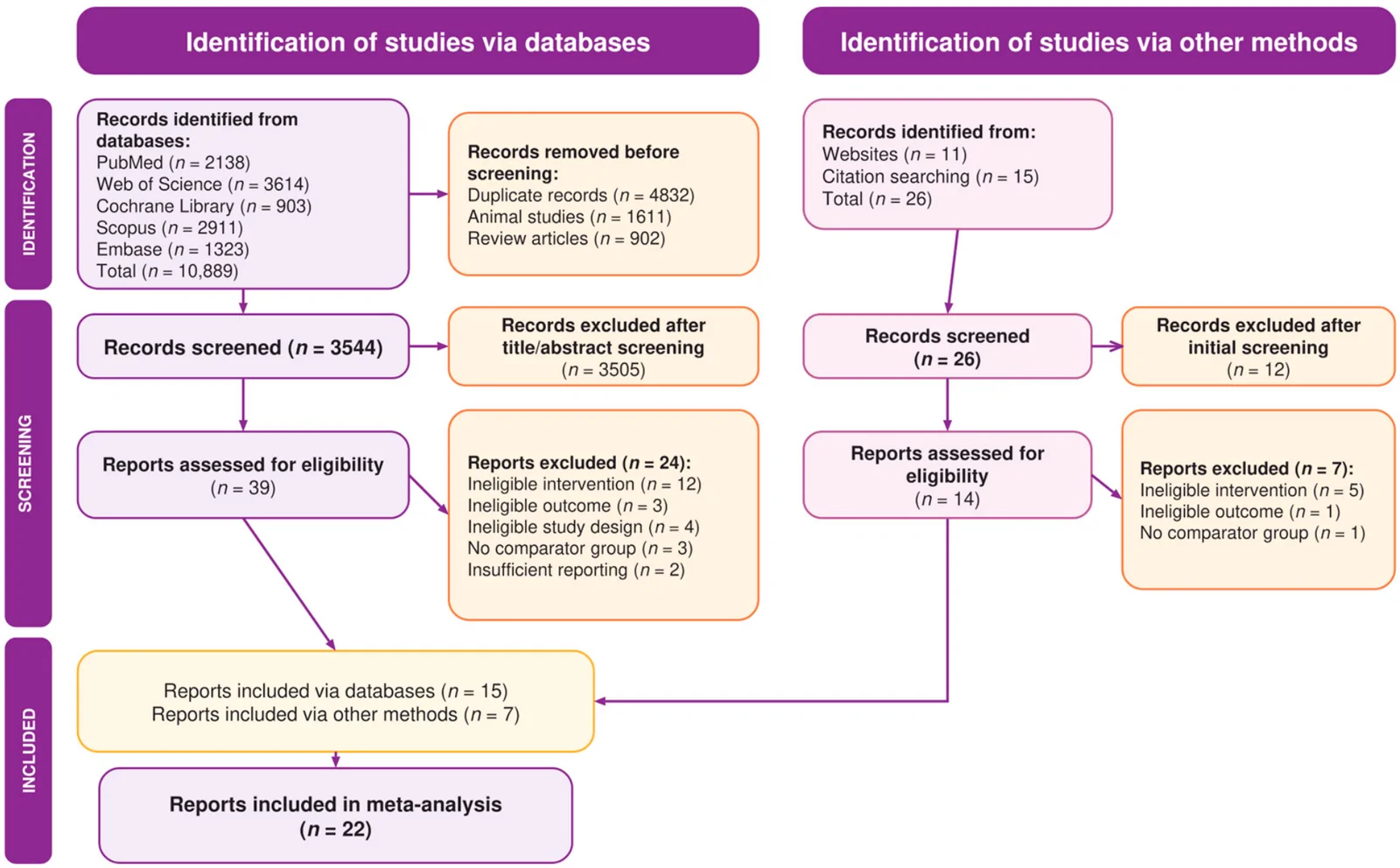
PRISMA 2020 flow diagram of the study-selection process.

The database searches identified 10,889 records, of which 3544 remained after removal of duplicates and other ineligible records. Thirty-nine database-derived reports underwent full-text assessment, and 15 met the eligibility criteria. Twenty-six additional records were identified through other methods; 14 were assessed in full, and seven were eligible. Thus, 53 reports were assessed in full, and 22 reports representing 20 independent controlled trials were included; 16 used randomized allocation, one used alternate allocation, two were non-randomized, and one did not report the allocation method (Figure 1).

### 3.2. Study Characteristics

The 22 reports were conducted in 11 countries, with sample sizes ranging from 11 to 124 participants per report. Group-level mean ages ranged from 16.2 to 33.1 years and mean BMIs from 20.4 to 37.6 kg/m^2^. Interventions generally lasted 8–24 weeks, involved two to seven sessions per week and 30–60 min per session, and were usually supervised. Modalities included walking, jogging or running, cycling, continuous and interval training, yoga, and Tai Chi. Table 1 summarizes the report-level characteristics; linked publications from the same participant cohort were analyzed as one independent study. All included reports were published before the renaming, and no eligible trial identified under either nomenclature had applied or reported the PMOS terminology.

### 3.3. Risk of Bias

**Figure 2:**
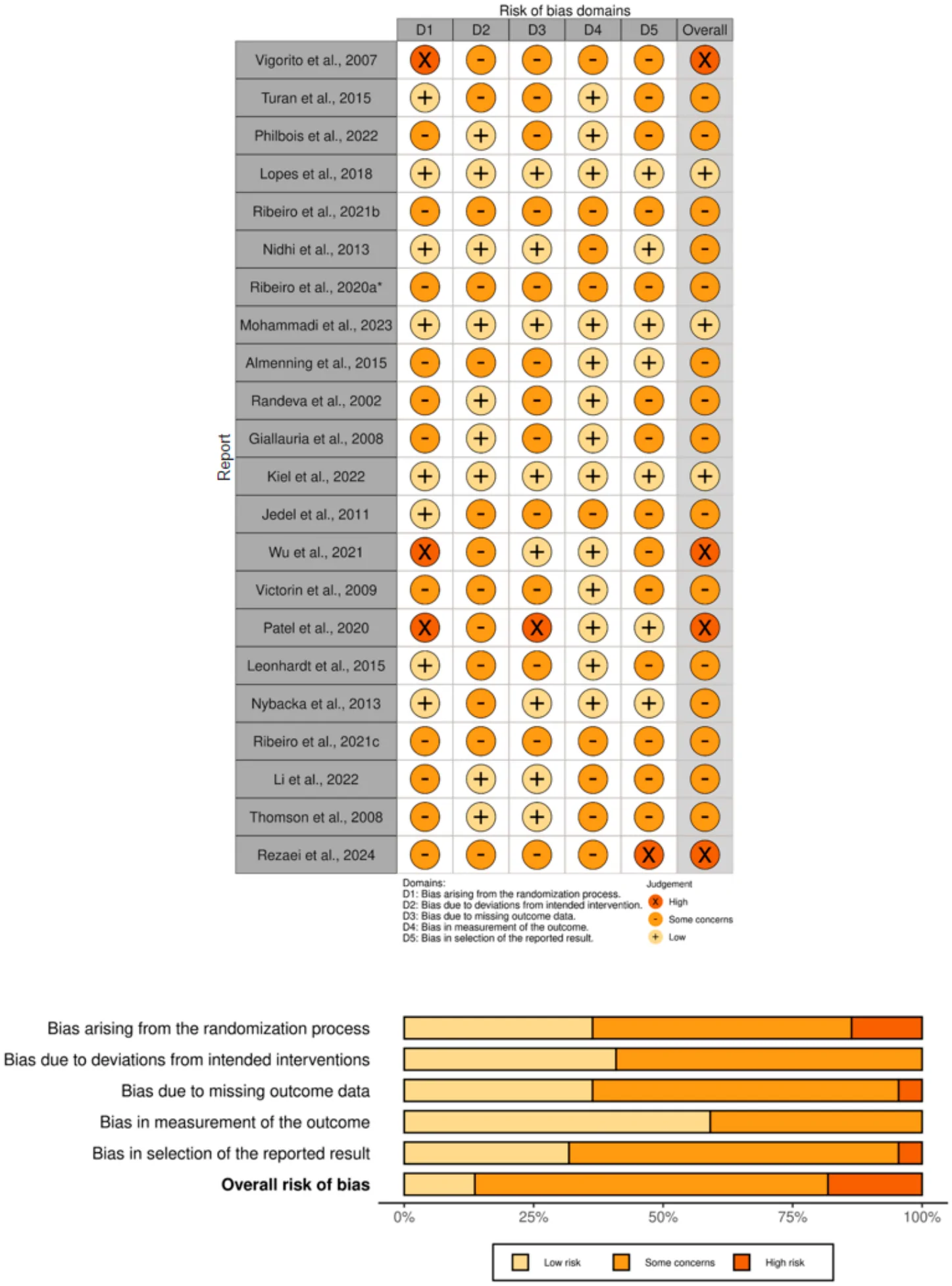
Risk-of-bias judgments across the 22 report-level assessments using the Cochrane Risk of Bias 2 tool. Report labels correspond to references [3,30,31,32,33,34,35,36,37,38,39,40,41,42,43,44,45,46,47,48,49,50]. Pale yellow circles (+) indicate low risk, orange circles (−) indicate some concerns, and dark orange circles (X) indicate high risk; the same colors identify judgments in the summary bars. Gray header and label backgrounds are used only to organize the display.

Across the 22 report-level assessments, three (13.6%) were judged at low risk of bias, 15 (68.2%) raised some concerns, and four (18.2%) were judged at high risk (Figure 2). Some concerns were most frequent for selection of the reported result (14/22, 63.6%), deviations from intended interventions (13/22, 59.1%), and missing outcome data (13/22, 59.1%). High-risk judgments occurred in the randomization process, missing outcome data, and selection of the reported result.

### 3.4. Anthropometric Outcomes

**Figure 3:**
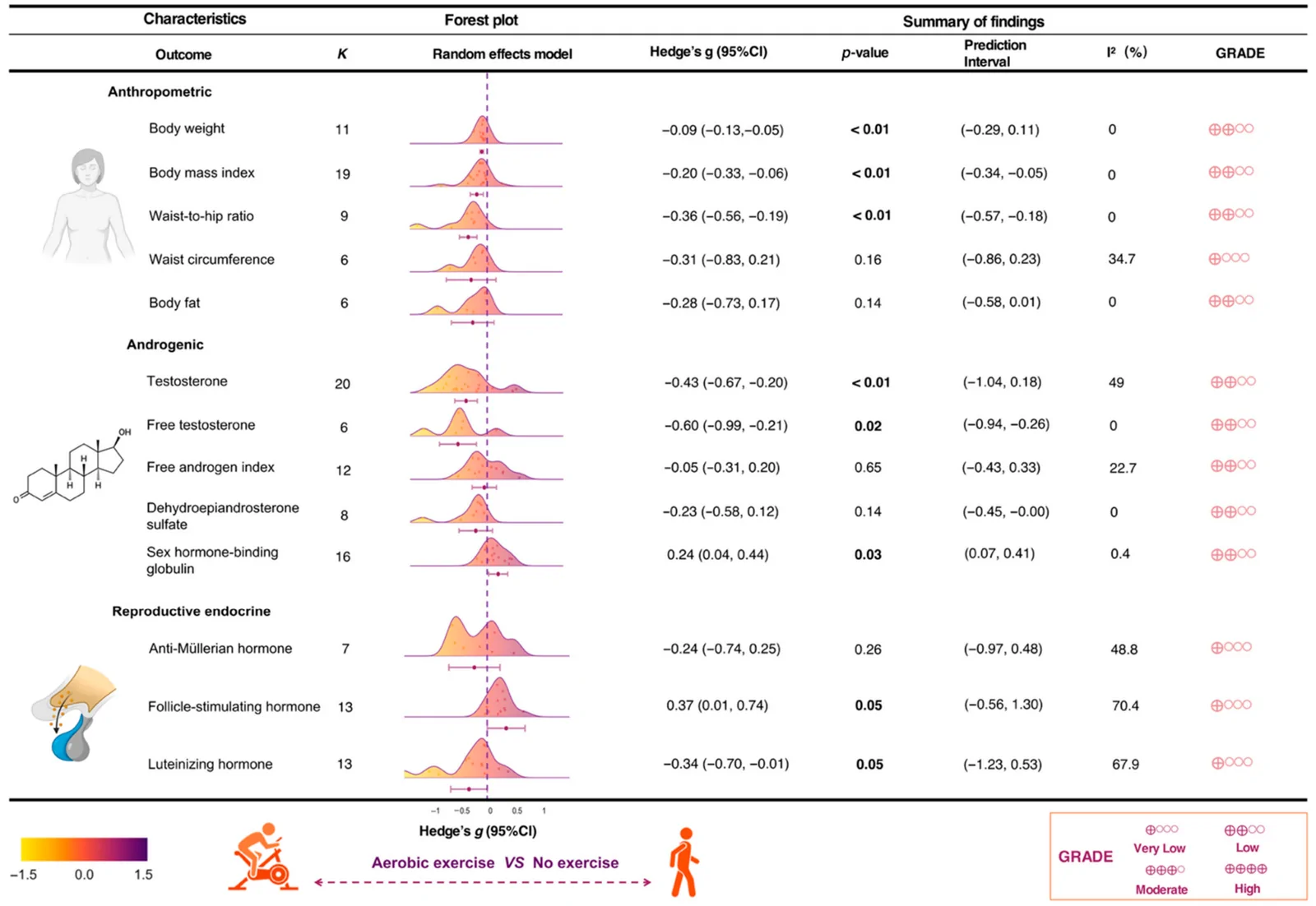
Pooled effects of aerobic or aerobic-oriented exercise and certainty of evidence for anthropometric, androgen-related, and reproductive endocrine outcomes in women with polycystic ovary syndrome. Note: Effects are Hedges’ g with 95% confidence intervals from random-effects models. Negative and positive values indicate lower and higher outcome levels, respectively, with exercise versus control. K, number of effect estimates; I^2^, heterogeneity; GRADE, Grading of Recommendations Assessment, Development, and Evaluation. Bold *p* values identify results reported at or below 0.05; estimates reported as *p* = 0.05 are interpreted as boundary-level findings. The prediction interval is written in full in the figure.

The pooled standardized estimate for body weight marginally favored exercise (k = 11; Hedges’ g = −0.09, 95% CI −0.13 to −0.05; *p* < 0.01; I^2^ = 0%; 95% prediction interval [PI] −0.29 to 0.11); however, its magnitude was trivial, and the 95% PI crossed the null. Exercise was associated with lower BMI (k = 19; g = −0.20, 95% CI −0.33 to −0.06; *p* < 0.01; I^2^ = 0%; 95% PI −0.34 to −0.05) and waist-to-hip ratio (k = 9; g = −0.36, 95% CI −0.56 to −0.19; *p* < 0.01; I^2^ = 0%; 95% PI −0.57 to −0.18) (Figure 3). No statistically detectable pooled effects were observed for waist circumference (k = 6; g = −0.31, 95% CI −0.83 to 0.21; *p* = 0.16; I^2^ = 34.7%; 95% PI −0.86 to 0.23) or body fat percentage (k = 6; g = −0.28, 95% CI −0.73 to 0.17; *p* = 0.14; I^2^ = 0%; 95% PI −0.58 to 0.01). Prediction intervals remained below zero for BMI and waist-to-hip ratio but crossed the null for waist circumference and body fat percentage.

Exploratory subgroup analyses for BMI found no statistically detectable difference by obesity status, exercise intensity, or exercise modality; categories represented by fewer than three independent trials were interpreted descriptively (Supplementary Table S8). Exercise modality was coded as running, cycling, combined training, or mind–body exercise, and pooled estimates did not differ detectably by modality for any eligible outcome (Supplementary Tables S8–S13). No examined study-level participant or prescription variable was associated with the BMI effect, and neither linear nor quadratic terms were detected for dose per session, weekly exercise time, or weekly dose (Supplementary Figure S1).

### 3.5. Androgen-Related Outcomes

Exercise was associated with lower total testosterone (k = 20; Hedges’ g = −0.43, 95% CI −0.67 to −0.20; *p* < 0.01; I^2^ = 49.0%; 95% PI −1.04 to 0.18) and free testosterone (k = 6; g = −0.60, 95% CI −0.99 to −0.21; *p* = 0.02; I^2^ = 0%; 95% PI −0.94 to −0.26) and with higher SHBG (k = 16; g = 0.24, 95% CI 0.04 to 0.44; *p* = 0.03; I^2^ = 0.4%; 95% PI 0.07 to 0.41) (Figure 3). No statistically detectable pooled effects were observed for FAI (k = 12; g = −0.05, 95% CI −0.31 to 0.20; *p* = 0.65; I^2^ = 22.7%; 95% PI −0.43 to 0.33) or DHEAS (k = 8; g = −0.23, 95% CI −0.58 to 0.12; *p* = 0.14; I^2^ = 0%; 95% PI −0.45 to 0.00).

Exploratory subgroup analyses for total testosterone, FAI, and SHBG found no statistically detectable differences by obesity status, exercise intensity, exercise modality, or supervision in eligible formal comparisons (Supplementary Tables S9–S11; Supplementary Figures S2–S4). Subgroup analyses were not conducted for free testosterone or DHEAS because the evidence base was too small.

**Figure 4:**
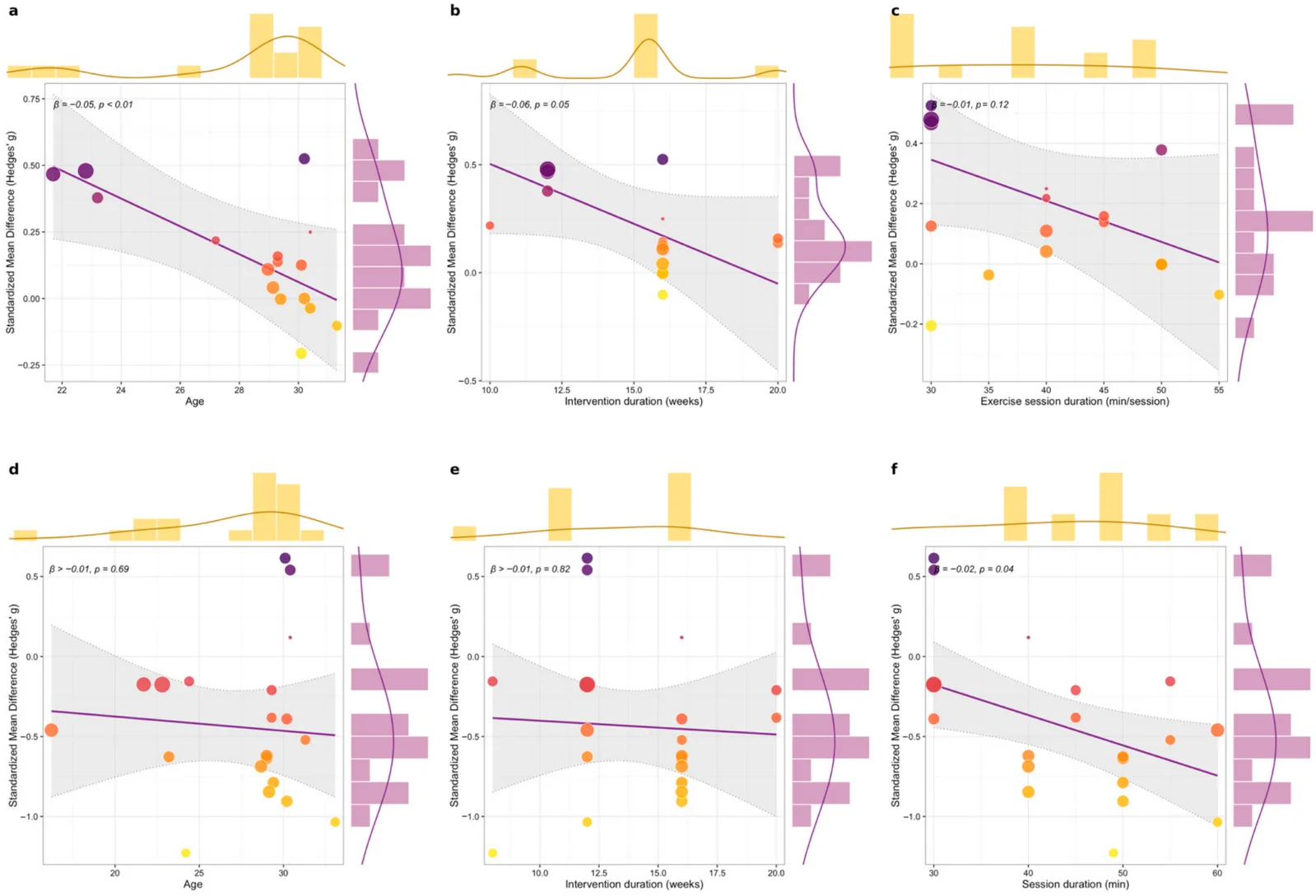
Meta-regression analyses of participant and exercise-prescription characteristics associated with the effects of exercise on total testosterone and sex hormone-binding globulin. Note: Panels (**a**–**c**) show associations of SHBG effect sizes with mean age, intervention duration, and session duration, respectively; panels (**d**–**f**) show the corresponding associations for total testosterone. Solid lines are fitted meta-regression slopes, and shaded bands are 95% confidence intervals. SHBG, sex hormone-binding globulin; β, meta-regression coefficient. Circles represent individual effect estimates. Marginal histograms and density curves show distributions of the moderator and effect estimates. Circle colors range from yellow for lower effect estimates to purple for higher effect estimates. The rounded regression coefficients displayed in panels (**c**) and (**f**) are β = −0.01 (*p* = 0.12) and β = −0.02 (*p* = 0.04), respectively.

**Figure 5:**
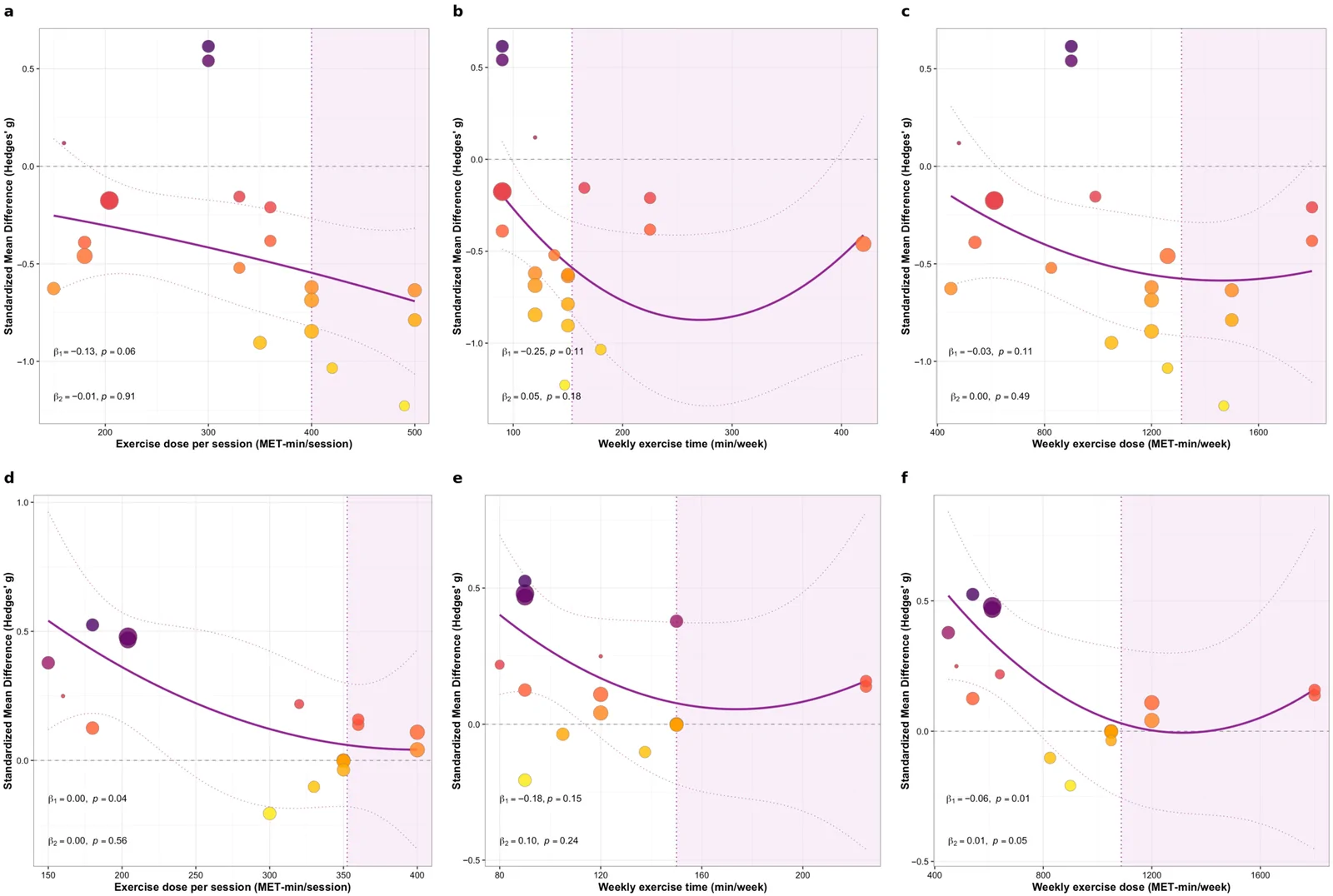
Dose–response meta-regression analyses of aerobic exercise volume and dose for testosterone and sex hormone-binding globulin. Note: Panels (**a**–**c**) show the associations of the effect on testosterone with exercise dose per session, weekly exercise time, and weekly exercise dose, respectively; panels (**d**–**f**) show the corresponding associations for sex hormone-binding globulin. Effect estimates are expressed as Hedges’ g. Solid curves represent the fitted quadratic meta-regression models, and dotted curves indicate the corresponding 95% confidence intervals. Each bubble represents an effect estimate, with bubble size proportional to its relative weight. Vertical dotted lines indicate the prespecified reference values, and shaded regions represent exposure levels above these values. β_1_ and β_2_ denote the linear and quadratic coefficients, respectively. MET, metabolic equivalent of task. These dose–response models are exploratory; observations are sparse at the extremes of the exposure range, and the fitted curves should not be used to identify a target exercise dose. Bubble colors range from yellow for lower effect estimates to purple for higher effect estimates. Horizontal gray dashed lines indicate no effect.

In exploratory univariable meta-regression, more negative total-testosterone effect sizes were associated with longer prescribed session duration (β = −0.02, *p* = 0.04). SHBG effect sizes were inversely associated with mean age (β = −0.05, *p* < 0.01) and showed a boundary-level inverse association with intervention duration (β = −0.06, reported *p* = 0.05). No other examined study-level participant or prescription variable was statistically associated with total testosterone or SHBG. These univariable, study-level models were unadjusted for multiplicity and are reported as hypothesis-generating (Figure 4 and Figure 5; Supplementary Figures S5–S7).

### 3.6. Reproductive Endocrine Outcomes

No statistically detectable pooled effect was observed for AMH (k = 7; Hedges’ g = −0.24, 95% CI −0.74 to 0.25; *p* = 0.26; I^2^ = 48.8%; 95% PI −0.97 to 0.48). The pooled estimates indicated higher FSH (k = 13; g = 0.37, 95% CI 0.01 to 0.74; reported *p* = 0.05; I^2^ = 70.4%; 95% PI −0.56 to 1.30) and lower LH (k = 13; g = −0.34, 95% CI −0.70 to −0.01; reported *p* = 0.05; I^2^ = 67.9%; 95% PI −1.23 to 0.53) (Figure 3). Both estimates lay at the conventional significance boundary, showed substantial heterogeneity, and had prediction intervals crossing the null.

Exploratory subgroup analyses did not identify differences in FSH or LH effects by obesity status, exercise intensity, or exercise modality (Supplementary Tables S12 and S13; Supplementary Figures S8 and S9). The examined study-level continuous moderators were not associated with either outcome (Supplementary Figures S10 and S11).

### 3.7. Small-Study Effects

**Figure 6:**
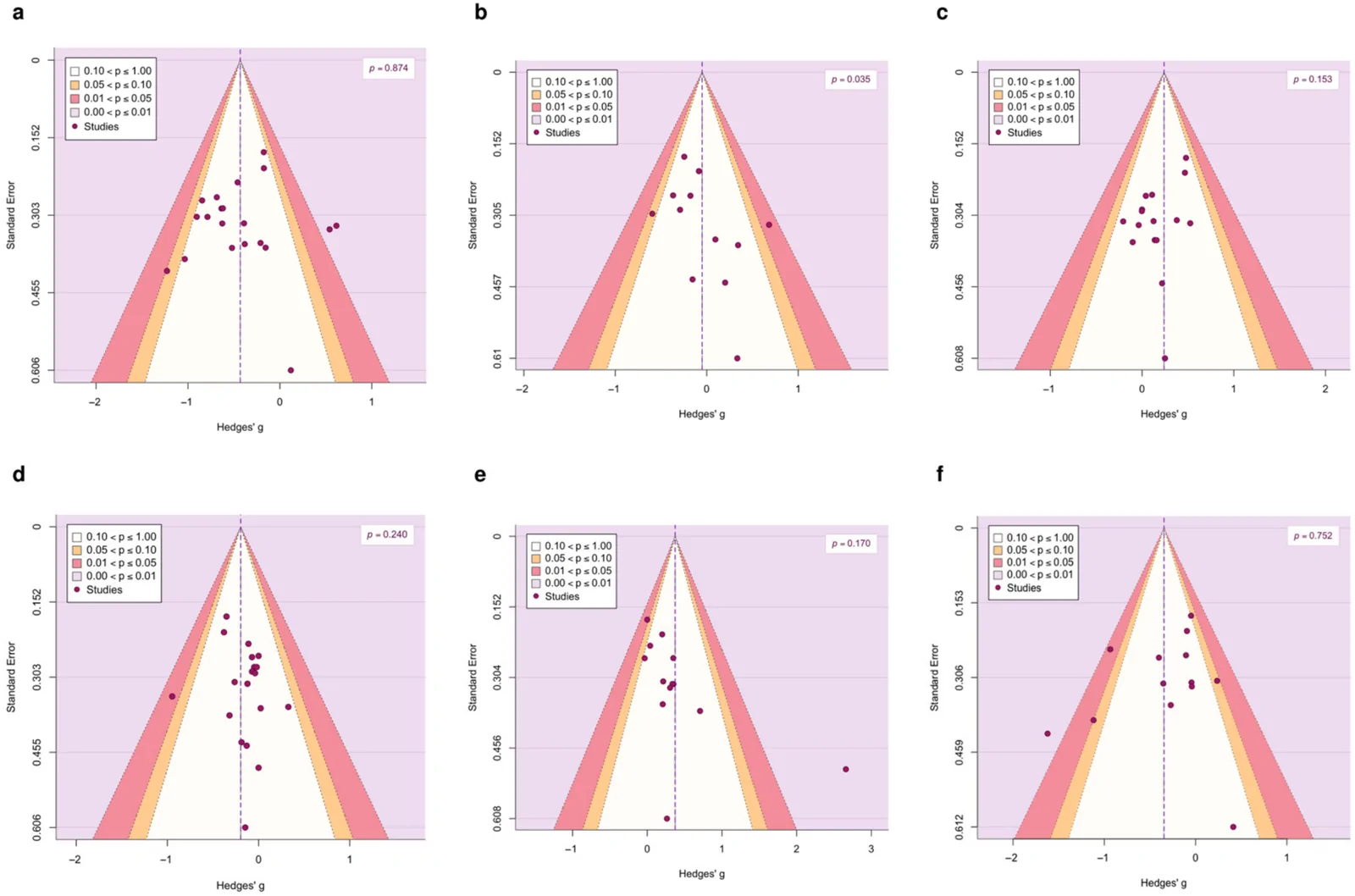
Contour-enhanced funnel plots for assessment of small-study effects. Note: Panels show (**a**) total testosterone, (**b**) free androgen index, (**c**) sex hormone-binding globulin, (**d**) body mass index, (**e**) follicle-stimulating hormone, and (**f**) luteinizing hormone. Dots are effect estimates, vertical dashed lines are pooled Hedges’ g values, and shaded regions show two-sided significance contours. The displayed *p* values are from cluster-robust Egger regression.

Contour-enhanced funnel plots were broadly symmetrical for most eligible outcomes. Cluster-robust Egger regression indicated a possible small-study-effect signal for FAI (*p* = 0.035), but not for total testosterone (*p* = 0.874), SHBG (*p* = 0.153), BMI (*p* = 0.240), FSH (*p* = 0.170), or LH (*p* = 0.752) (Figure 6). Outcomes represented by fewer than 10 independent studies were examined descriptively (Supplementary Figure S12). The FAI result was one of several tests and was close to the minimum information threshold; it was not interpreted as proof of publication bias.

### 3.8. Sensitivity Analyses

**Figure 7:**
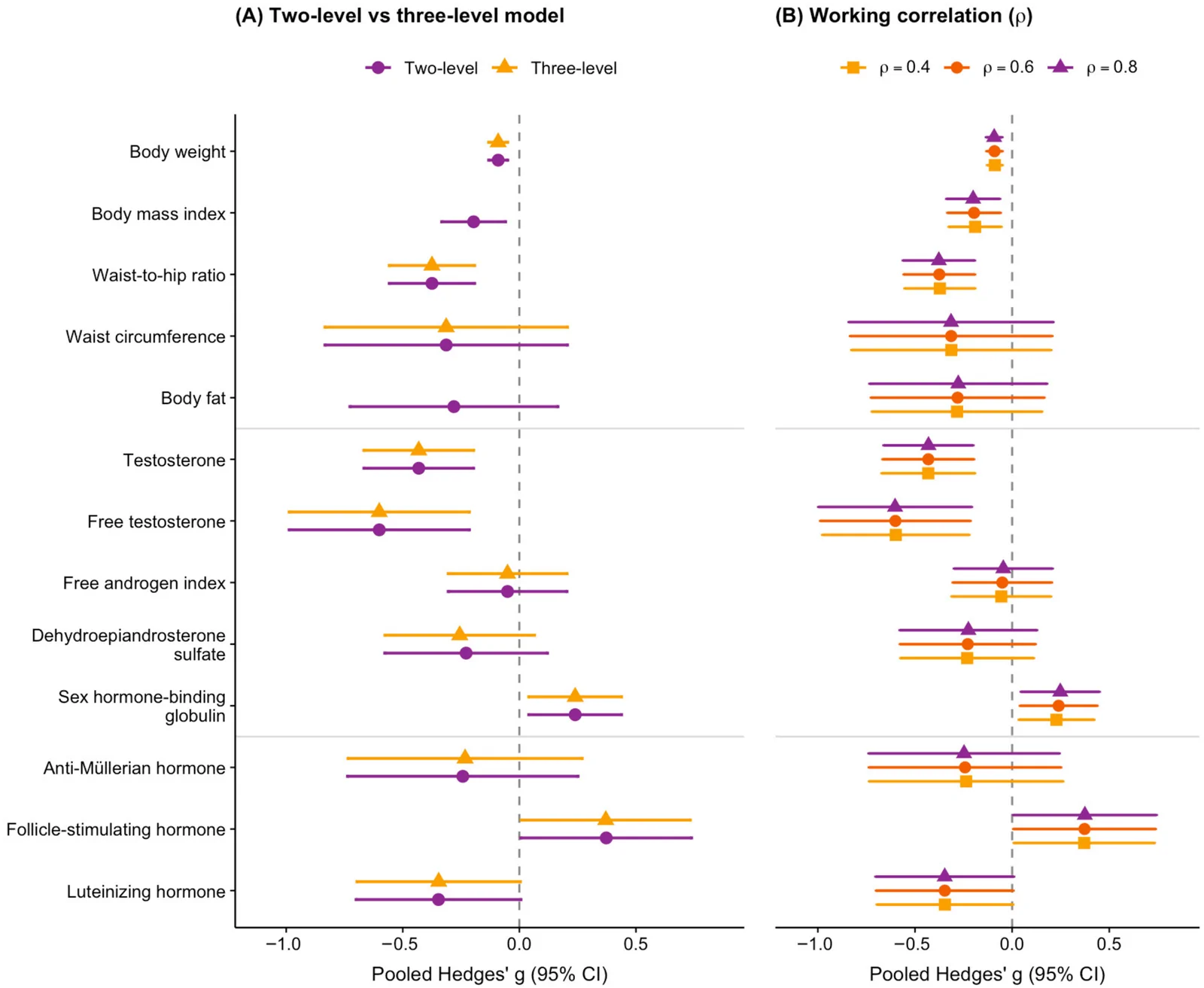
Sensitivity analyses across alternative model structures and working within-study correlations. Note: Panel (**A**) compares pooled estimates from two- and three-level random-effects models. Panel (**B**) compares estimates using working within-study correlations of ρ = 0.40, 0.60, and 0.80. Symbols indicate Hedges’ g, horizontal lines are 95% confidence intervals, and vertical dashed lines show the null.

Two- and three-level models produced comparable estimates, and varying the assumed within-study correlation from ρ = 0.40 to 0.80 caused negligible changes in direction, magnitude, or precision (Figure 7). Leave-one-study-out analyses did not materially change the conclusions for anthropometric outcomes, total testosterone, FAI, DHEAS, or AMH. Statistical significance changed after exclusion of individual studies for free testosterone, SHBG, FSH, and LH, although the direction of effect was unchanged (Supplementary Figures S13–S15).

### 3.9. Certainty of Evidence

Certainty ranged from low to very low (Supplementary Table S14). Evidence was rated low for body weight, BMI, waist-to-hip ratio, body fat percentage, and all androgen-related outcomes, and very low for waist circumference, AMH, FSH, and LH. Downgrading primarily reflected risk of bias and imprecision; no outcome was supported by moderate- or high-certainty evidence. Indirectness was not rated down because certainty was assessed for the prespecified biochemical and anthropometric outcomes; these ratings should not be read as certainty about ovarian function or reproductive outcomes.

## 4. Discussion

This synthesis suggests a selective rather than uniform response to aerobic or aerobic-oriented exercise. The pooled body-weight estimate was trivial, and its prediction interval crossed the null. Pooled estimates favored exercise for BMI, waist-to-hip ratio, total testosterone, free testosterone, and SHBG, whereas waist circumference, body fat percentage, FAI, DHEAS, and AMH showed no clear pooled change. The FSH and LH estimates were statistically borderline, heterogeneous, and sensitive to individual studies; inference for free testosterone and SHBG also changed after individual-study omission, although the direction of effect did not. Together with low or very-low certainty across outcomes, this pattern is more consistent with outcome-specific biochemical and anthropometric differences than with a generalized normalization of the PCOS endocrine profile. Total testosterone, free testosterone, and SHBG are circulating biochemical markers rather than measures of ovarian function, and the differences observed here are therefore not direct evidence of improved ovulation, fertility, or other reproductive outcomes.

Previous reviews provide evidence across different exercise modalities, intervention contrasts, and endocrine outcomes [4,5,6,7]. The present review complements these syntheses by evaluating individual endocrine markers alongside anthropometric outcomes and interpreting pooled estimates together with prediction intervals, sensitivity analyses, and certainty of evidence. Differences in the intervention contrast, the eligible comparator, outcome availability, effect-size construction, and the treatment of statistically dependent estimates may partly account for variation among reviews. This approach makes the uncertainty around favorable pooled estimates explicit. For example, the pooled estimates for free testosterone and SHBG favored exercise, but inference was sensitive to individual-study omission, and certainty was low. The heterogeneous intervention scope limits attribution to conventional aerobic training alone and does not establish superiority over other exercise modalities. Modality comparisons did not detect differences between mind–body and conventional endurance interventions, but the mind–body evidence base was small, and this is not evidence of equivalence.

SHBG warrants interpretation separately from circulating testosterone. It is synthesized predominantly in the liver and may reflect metabolic and hepatic regulation. In women with PCOS, cross-sectional observations coupled with HepG2 experiments linked greater oxidative stress to lower SHBG and showed reduced HNF-4α-dependent SHBG expression under oxidized-lipoprotein exposure [51]. This evidence makes a hepatic metabolic pathway biologically plausible, but the trials in the present review did not test oxidative stress, hepatic fat, insulin sensitivity, or HNF-4α as mediators. The coexistence of higher pooled SHBG with a null FAI estimate is also not intrinsically contradictory: FAI is a ratio, the contributing trial subsets differed, and changes in both testosterone and SHBG are sensitive to assay and sampling conditions. The present findings therefore indicate an SHBG response signal, not proof of reduced ovarian androgen production or restored reproductive function.

The exploratory moderator analyses do not define an endocrine-targeted exercise dose. Associations with participant age, intervention duration, or session duration were estimated from between-trial contrasts rather than from randomized within-trial comparisons, were unadjusted for multiplicity, and may reflect correlated intervention or population characteristics; they describe how trials differed from one another and cannot be transferred to individual patients. Evidence from an active-comparator trial [52], a network meta-analysis [53], and an observational substitution analysis [54] addresses related but distinct questions and cannot validate a dose threshold. Prospective trials with prespecified dose contrasts are required before any clinically actionable inference.

The exploratory moderator results likewise describe study-level variation rather than patient-level treatment selection. No categorical subgroup variable differentiated the pooled effects, while isolated associations linked total testosterone with prescribed session duration and SHBG with mean age and intervention duration. Failure to detect a subgroup difference is not evidence that PCOS phenotypes respond identically. Data-driven work has identified hyperandrogenic, obesity-related, high-SHBG, and high-LH/AMH phenotypes with different clinical trajectories [55]; metabolic dysfunction has also been documented in adolescents without excess adiposity [56], and hyperandrogenic phenotypes have been associated with poorer pregnancy and live-birth outcomes after accounting for BMI [57]. These studies show that BMI-only stratification is coarse, but none demonstrates that phenotype modifies the response to exercise, and none of the present analyses was designed to evaluate the multisystem characterization underlying the renaming.

Clinical interpretation is further limited by the distance between biochemical markers and patient-important reproductive outcomes. In a secondary, within-intervention analysis of a multicomponent lifestyle program, resumption of ovulation was associated with change in an adrenal androgen metabolite, whereas SHBG did not clearly distinguish women who did and did not resume ovulation [58]. In adults with overweight or obesity, adding exercise to a hypocaloric diet did not yield an additional pooled change in testosterone, estradiol, or SHBG; this non-PCOS evidence is indirect but illustrates that metabolic and sex-hormone responses need not move in parallel [59]. Measurement also matters: a diagnostic meta-analysis found that androgen assays differ in accuracy and that liquid chromatography–tandem mass spectrometry generally performs better than direct immunoassays for several measures [60]. Standardization as Hedges’ g aligns numerical scales but cannot remove differences in assay platform, menstrual-cycle timing, medication restrictions, or sampling context. Because menstrual regularity, ovulation, pregnancy, live birth, hirsutism, and quality of life were not synthesized, the observed hormone changes should not be interpreted as evidence of fertility benefit or symptom improvement.

The review’s strengths include prospective registration, an outcome-specific synthesis of anthropometric and endocrine outcomes, explicit handling of dependent estimates, cluster-robust small-sample inference, prediction intervals, structured exercise-dose coding, and outcome-level certainty assessment. Important limitations remain: many trials were small and short; risk of bias was common; and diagnostic criteria, exercise prescriptions, outcome assays, sampling conditions, and adherence reporting varied. No included trial was designed or reported within the PMOS framework, and the findings should be read as evidence in women meeting established PCOS diagnostic criteria. Exposure reflected prescribed rather than achieved exercise, and the univariable study-level moderator and dose–response analyses were unadjusted for multiplicity and vulnerable to ecological confounding and influential observations. Sparse evidence for several outcomes also constrained precision, prediction, funnel-plot interpretation, and sensitivity analysis. These limitations do not alter the general-health rationale for physical activity in comprehensive PCOS care [2], but they separate that recommendation from an endocrine-targeted prescription. Future trials should compare clearly separated doses and intensities, quantify achieved exercise, standardize endocrine assays and menstrual-cycle timing, prespecify metabolic mediators, and measure reproductive and patient-reported outcomes alongside biomarkers; collaborative participant-level datasets would be needed to examine phenotype-specific response.

## 5. Conclusions

Pooled standardized estimates favored aerobic or aerobic-oriented exercise for selected anthropometric and androgen-related markers in women with PCOS, but certainty was low or very low. The body-weight effect was trivial, and inference for free testosterone and SHBG was sensitive to individual-study omission. The evidence does not establish a clinically important reduction in body weight, a modality-specific effect, an endocrine-targeted exercise dose, or reproductive benefit.

## Figures and Tables

**Table 1:** Characteristics of the included reports and exercise interventions.

Study/Report	Country/Design	Sample, *n*	Exercise Protocol	Outcomes
Vigorito et al. 2007 [30]	Italy; RCT	EX 45; CON 45	Cycling; 60–70% VO_2_max; 12 wk; 3 sessions/wk; 30 min/session	T, LH, FSH, SHBG, FAI, BMI, WC, WHR
Turan et al. 2015 [31]	Turkey; RCT; mixed aerobic-resistance intervention	EX 14; CON 16	Combined training; 65–70% HRmax; 8 wk; 3 sessions/wk; 55 min/session	T, LH, FSH, FT, BMI, WC, HC
Philbois et al. 2022 [32]	Brazil; RCT	MICT 25; HIIT 25; CON 25	Running (MICT); running (HIIT); MICT 70–80% HRR; HIIT 85–90% HRR; 16 wk; 3 sessions/wk; MICT 50, HIIT 40 min/session	T, Weight, BMI
Lopes et al. 2018 [33]	Brazil; RCT	CAT 23; IAT 22; CON 24	Running (CAT); running (IAT); CAT 75–80% HRmax; IAT 85–90% HRmax; 16 wk; 3 sessions/wk; 50 min/session	T, SHBG, FAI, Weight, BMI, WHR
Ribeiro et al. 2021 (quality-of-life report) [34]	Brazil; RCT	CAT 28; IAT 29; CON 30	Treadmill running (CAT); treadmill running (IAT); CAT 75–80% HRmax; IAT 85–90% HRmax; 16 wk; 3 sessions/wk; 40 min/session	T, BMI, WC, WHR, HC
Nidhi et al. 2013 [35]	India; Active-controlled RCT	Yoga 37; conventional exercise 35	Yoga; NR; 12 wk; 7 sessions/wk; 60 min/session	T, AMH, LH, FSH, Weight, BMI
Ribeiro et al. 2020 (hormonal/body-composition report) [36]	Brazil; RCT	CAT 28; IAT 29; CON 30	Treadmill running (CAT); treadmill running (IAT); CAT 75–80% HRmax; IAT 85–90% HRmax; 16 wk; 3 sessions/wk; 40 min/session	T, LH, FSH, SHBG, FAI, Weight, BMI, WC, WHR, HC, BF%
Mohammadi et al. 2023 [37]	Iran; RCT	HIIT 14; CON 14	Running (HIIT); 100% MAV; 8 wk; 3 sessions/wk; 49 min/session	T, Weight, BMI, WHR, BF%, VFA
Almenning et al. 2015 [38]	Norway; RCT	HIIT 8; CON 9	HIIT using running, cycling, or walking; 90–95% HRmax; 10 wk; 2 sessions/wk; 40 min/session	AMH, SHBG, FAI, DHEAS, Weight, BMI, WC, BF%, VFA, FM, FFM
Randeva et al. 2002 [39]	United Kingdom; open longitudinal; non-randomized	EX 12; CON 9	Brisk walking; NR; 24 wk; 4 sessions/wk; 30 min/session	FAI, Weight, WHR
Giallauria et al. 2008 [40]	Italy; patient-choice; non-randomized	EX 62; CON 62	Cycling; 60–70% VO_2_max; 12 wk; 3 sessions/wk; 30 min/session	T, LH, FSH, SHBG, FAI, DHEAS, Weight, WHR
Kiel et al. 2022 [41]	Norway; three-arm RCT	HV-HIIT 20; LV-HIIT 18; CON 20 analyzed (20/21/23 randomized)	Running (HV-HIIT); running (LV-HIIT); HV-HIIT 90–95% HRmax; LV-HIIT NR; 16 wk: 3 sessions/wk (HV 30, LV 35 min); 52-wk follow-up: 2 sessions/wk	T, AMH, SHBG, FAI
Jedel et al. 2011 [42]	Sweden; RCT	EX 30; CON 15	Self-selected running, cycling, or walking; HR ≥ 120 beats/min; 16 wk; 3 sessions/wk; 30 min/session	T, LH, FSH, SHBG, FT, DHEAS, BMI
Wu et al. 2021 [43]	China; alternate allocation; quasi-randomized	EX 19; CON 19	Cycling; NR; 12 wk; 4 sessions/wk; 60 min/session	T, AMH, LH, FSH, DHEAS, BMI
Stener-Victorin et al. 2009 [44]	Sweden; RCT	EX 5; CON 6	Self-selected running, cycling, or walking; faster than usual walking (low intensity); 16 wk; 3 sessions/wk; 40 min/session	T, LH, FSH, SHBG, FAI, FT, DHEAS, BMI, WHR
Patel et al. 2020 [45]	USA; RCT	Yoga 18; CON 9	Yoga; NR; 12 wk; 3 sessions/wk; 60 min/session	FT, DHEAS, BMI, WHR
Leonhardt et al. 2015 [46]	Sweden; RCT	EX 30; CON 15	Self-selected running, cycling, or walking; faster than usual walking (low intensity); 16 wk; 3 sessions/wk; 30 min/session	AMH
Nybacka et al. 2013 [47]	Sweden; three-arm RCT; no inactive control	EX 19; CON 19	Self-selected running, cycling, or walking; moderate intensity; 16 wk; 2.5 sessions/wk; 55 min/session	T, AMH, LH, FSH, SHBG, FT, BMI, BF%
Ribeiro et al. 2021 (telomere report) [3]	Brazil; RCT	CAT 28; IAT 29; CON 30	Treadmill running (CAT); treadmill running (IAT); CAT 75–80% HRmax; IAT 85–90% HRmax; 16 wk; 3 sessions/wk; 40 min/session	T, LH, FSH, SHBG, FAI, Weight, BMI, WC, WHR, HC, BF%
Li et al. 2022 [48]	China; active-controlled RCT	Tai Chi 24; self-monitored exercise 18	Tai Chi; low intensity; 12 wk; 3 sessions/wk; 50 min/session	T, LH, FSH, SHBG, DHEAS, Weight, BMI, WHR
Thomson et al. 2008 [49]	Australia; three-arm RCT; common diet background	Diet + aerobic 18; diet-only 14	Walking/jogging; 75–80% HRmax; 20 wk; 5 sessions/wk; 45 min/session	T, SHBG, FAI, Weight, WC, BF%, FM, FFM
Rezaei et al. 2024 [50]	Iran; semi-experimental; allocation unclear	EX 15; CON 15	Walking/jogging; 70–80% HRmax; 12 wk; 3 sessions/wk; 60 min/session	T, LH, FSH

Note: Linked reports from the same trial were clustered at the independent-study level. AMH, anti-Müllerian hormone; BF%, body fat percentage; BMI, body mass index; CAT, continuous aerobic training; CON, control; DHEAS, dehydroepiandrosterone sulfate; EX, exercise; FAI, free androgen index; FFM, fat-free mass; FM, fat mass; FSH, follicle-stimulating hormone; FT, free testosterone; HC, hip circumference; HIIT, high-intensity interval training; HRmax, maximal heart rate; HRR, heart-rate reserve; IAT, intermittent aerobic training; LH, luteinizing hormone; MICT, moderate-intensity continuous training; NR, not reported; SHBG, sex hormone-binding globulin; T, total testosterone; VFA, visceral fat area; VO_2_max, maximal oxygen uptake; WC, waist circumference; WHR, waist-to-hip ratio. wk, week(s).

## Data Availability

The original contributions presented in this study are included in the article and Supplementary Materials. Further inquiries can be directed to the corresponding authors.

## Supplementary Materials

The following supporting information can be downloaded at https://www.mdpi.com/article/10.3390/metabo16100745/s1, Supplementary Tables S1 and S2: PRISMA 2020 checklists; Supplementary Tables S3–S7: Database search strategies; Supplementary Tables S8–S13: Subgroup analyses; Supplementary Figures S1–S11: Exploratory subgroup, meta-regression, and dose–response analyses; Supplementary Figure S12: Additional funnel plots; Supplementary Figures S13–S15: Leave-one-study-out analyses; Supplementary Table S14: GRADE certainty assessment.
