# Supplementary material for: Aerobic and Aerobic-Oriented Exercise for Anthropometric, Androgen-Related, and Reproductive Endocrine Outcomes in Polyendocrine Metabolic Ovarian Syndrome (Polycystic Ovary Syndrome): A Systematic Review and Meta-Analysis
Source: Metabolites. 2026 Oct 3;16(10):745. doi: 10.3390/metabo16100745 (PMC13706977; doi:10.3390/metabo16100745)
Supplement: Supplementary file 1 [file metabolites-16-00745-s001.zip › metabolites-4542109-supplementary.pdf]

SUPPLEMENTARY MATERIAL

Aerobic and Aerobic-Oriented Exercise for Anthropometric, Androgen-Related, and Reproductive Endocrine Outcomes in Polyendocrine Metabolic Ovarian Syndrome: A Systematic Review and Meta-Analysis

Lanxin Qiu[1,2,‡], Yunzhi Emma Huang[3 ‡], Mingnan Zhuang[4,‡], Juntao Kan[3,4,*] and Zhengqi Qiu[1,3,*]

Contents

Part 1. PRISMA 2020 reporting checklists
Part 2. Database search strategies
Part 3. Numerical subgroup analysis results
Part 4. Graphical subgroup, meta-regression, and dose-response results
Part 5. Publication bias assessment
Part 6. Sensitivity analyses
Part 7. GRADE certainty of evidence

Part 1. PRISMA 2020 Reporting Checklists

Supplementary Table S1. PRISMA 2020 for Abstracts checklist.

Section and topic	Item	Checklist item	Reported (Yes/No)
**TITLE**			
Title	1	Identify the report as a systematic review.	Yes
**BACKGROUND**			
Objectives	2	Provide an explicit statement of the main objective(s) or question(s) the review addresses.	Yes
**METHODS**			
Eligibility criteria	3	Specify the inclusion and exclusion criteria for the review.	Yes
Information sources	4	Specify the information sources (e.g. databases, registers) used to identify studies and the date when each was last searched.	No
Risk of bias	5	Specify the methods used to assess risk of bias in the included studies.	No
Synthesis of results	6	Specify the methods used to present and synthesise results.	Yes
**RESULTS**			
Included studies	7	Give the total number of included studies and participants and summarise relevant characteristics of studies.	No
Synthesis of results	8	Present results for main outcomes, preferably indicating the number of included studies and participants for each. If meta-analysis was done, report the summary estimate and confidence/credible interval. If comparing groups, indicate the direction of the effect (i.e. which group is favoured).	Yes
**DISCUSSION**			
Limitations of evidence	9	Provide a brief summary of the limitations of the evidence included in the review (e.g. study risk of bias, inconsistency and imprecision).	Yes
Interpretation	10	Provide a general interpretation of the results and important implications.	Yes
**OTHER**			
Funding	11	Specify the primary source of funding for the review.	No
Registration	12	Provide the register name and registration number.	No

Supplementary Table S2. PRISMA 2020 checklist.

Section and topic	Item	Checklist item	Location where item is reported
**TITLE**			
Title	1	Identify the report as a systematic review.	Title
**ABSTRACT**			
Abstract	2	See the PRISMA 2020 for Abstracts checklist.	Abstract; Supplementary Table S1
**INTRODUCTION**			
Rationale	3	Describe the rationale for the review in the context of existing knowledge.	Introduction
Objectives	4	Provide an explicit statement of the objective(s) or question(s) the review addresses.	Introduction
**METHODS**			
Eligibility criteria	5	Specify the inclusion and exclusion criteria for the review and how studies were grouped for the syntheses.	Methods: eligibility criteria
Information sources	6	Specify all databases, registers, websites, organisations, reference lists and other sources searched or consulted to identify studies. Specify the date when each source was last searched or consulted.	Methods: information sources and literature search
Search strategy	7	Present the full search strategies for all databases, registers and websites, including any filters and limits used.	Supplementary Tables S3–S7
Selection process	8	Specify the methods used to decide whether a study met the inclusion criteria of the review, including how many reviewers screened each record and each report retrieved, whether they worked independently, and if applicable, details of automation tools used in the process.	Methods: study selection
Data collection process	9	Specify the methods used to collect data from reports, including how many reviewers collected data from each report, whether they worked independently, any processes for obtaining or confirming data from study investigators, and if applicable, details of automation tools used in the process.	Methods: data extraction
Data items	10a	List and define all outcomes for which data were sought. Specify whether all results that were compatible with each outcome domain in each study were sought (e.g. for all measures, time points, analyses), and if not, the methods used to decide which results to collect.	Methods: outcomes
Data items	10b	List and define all other variables for which data were sought (e.g. participant and intervention characteristics, funding sources). Describe any assumptions made about any missing or unclear information.	Methods: data extraction and moderator coding
Study risk of bias assessment	11	Specify the methods used to assess risk of bias in the included studies, including details of the tool(s) used, how many reviewers assessed each study and whether they worked independently, and if applicable, details of automation tools used in the process.	Methods: risk-of-bias assessment

Section and topic	Item	Checklist item	Location where item is reported
Effect measures	12	Specify for each outcome the effect measure(s) (e.g. risk ratio, mean difference) used in the synthesis or presentation of results.	Methods: effect-size calculation
Synthesis methods	13a	Describe the processes used to decide which studies were eligible for each synthesis (e.g. tabulating the study intervention characteristics and comparing against the planned groups for each synthesis (item #5)).	Methods: eligibility and synthesis groups
Synthesis methods	13b	Describe any methods required to prepare the data for presentation or synthesis, such as handling of missing summary statistics, or data conversions.	Methods: data preparation
Synthesis methods	13c	Describe any methods used to tabulate or visually display results of individual studies and syntheses.	Methods: data presentation
Synthesis methods	13d	Describe any methods used to synthesize results and provide a rationale for the choice(s). If meta-analysis was performed, describe the model(s), method(s) to identify the presence and extent of statistical heterogeneity, and software package(s) used.	Methods: statistical synthesis
Synthesis methods	13e	Describe any methods used to explore possible causes of heterogeneity among study results (e.g. subgroup analysis, meta-regression).	Methods: subgroup and meta-regression analyses
Synthesis methods	13f	Describe any sensitivity analyses conducted to assess robustness of the synthesized results.	Methods: sensitivity analyses
Reporting bias assessment	14	Describe any methods used to assess risk of bias due to missing results in a synthesis (arising from reporting biases).	Methods: reporting-bias assessment
Certainty assessment	15	Describe any methods used to assess certainty (or confidence) in the body of evidence for an outcome.	Methods: certainty assessment
**RESULTS**			
Study selection	16a	Describe the results of the search and selection process, from the number of records identified in the search to the number of studies included in the review, ideally using a flow diagram.	Results: search results; Figure 1
Study selection	16b	Cite studies that might appear to meet the inclusion criteria, but which were excluded, and explain why they were excluded.	Figure 1
Study characteristics	17	Cite each included study and present its characteristics.	Table 1
Risk of bias in studies	18	Present assessments of risk of bias for each included study.	Results: risk of bias; Figure 2
Results of individual studies	19	For all outcomes, present, for each study: (a) summary statistics for each group (where appropriate) and (b) an effect estimate and its precision (e.g. confidence/credible interval), ideally using structured tables or plots.	Not reported

Section and topic	Item	Checklist item	Location where item is reported
Results of syntheses	20a	For each synthesis, briefly summarise the characteristics and risk of bias among contributing studies.	Results; Table 1 and Figure 2
Results of syntheses	20b	Present results of all statistical syntheses conducted. If meta-analysis was done, present for each the summary estimate and its precision (e.g. confidence/credible interval) and measures of statistical heterogeneity. If comparing groups, describe the direction of the effect.	Results; Figure 3 and Supplementary Material
Results of syntheses	20c	Present results of all investigations of possible causes of heterogeneity among study results.	Results; Supplementary Tables S8-S13 and Figures S1-S11
Results of syntheses	20d	Present results of all sensitivity analyses conducted to assess the robustness of the synthesized results.	Results; Figure 7 and Supplementary Figures S13-S15
Reporting biases	21	Present assessments of risk of bias due to missing results (arising from reporting biases) for each synthesis assessed.	Results: reporting biases; Figure 6 and Supplementary Figure S12
Certainty of evidence	22	Present assessments of certainty (or confidence) in the body of evidence for each outcome assessed.	Results; Supplementary Table S14
**DISCUSSION**			
Discussion	23a	Provide a general interpretation of the results in the context of other evidence.	Discussion
Discussion	23b	Discuss any limitations of the evidence included in the review.	Discussion: limitations of the evidence
Discussion	23c	Discuss any limitations of the review processes used.	Discussion: limitations of the review process
Discussion	23d	Discuss implications of the results for practice, policy, and future research.	Discussion and Conclusions
**OTHER INFORMATION**			

Section and topic	Item	Checklist item	Location where item is reported
Registration and protocol	24a	Provide registration information for the review, including register name and registration number, or state that the review was not registered.	Methods: protocol and registration
Registration and protocol	24b	Indicate where the review protocol can be accessed, or state that a protocol was not prepared.	Methods: protocol and registration
Registration and protocol	24c	Describe and explain any amendments to information provided at registration or in the protocol.	No amendments to the registered protocol
Support	25	Describe sources of financial or non-financial support for the review, and the role of the funders or sponsors in the review.	Funding; funder role reported
Competing interests	26	Declare any competing interests of review authors.	Conflicts of Interest
Availability of data, code and other materials	27	Report which of the following are publicly available and where they can be found: template data collection forms; data extracted from included studies; data used for all analyses; analytic code; any other materials used in the review.	Data Availability Statement

Part 2. Database Search Strategies

Searches used two concepts: (1) PMOS/PCOS and related historical terminology and (2) aerobic or aerobic-oriented exercise. Outcome terms were not included in the final searches, consistent with the methods reported in the main manuscript.

Supplementary Table S3. Search strategy for PubMed/MEDLINE.

Search line	Search strategy
#1 Population	"Polycystic Ovary Syndrome"[Mesh] OR "polyendocrine metabolic ovarian syndrome"[tiab] OR PMOS[tiab] OR "polycystic ovary syndrome"[tiab] OR "polycystic ovarian syndrome"[tiab] OR PCOS[tiab] OR "Stein-Leventhal syndrome"[tiab] OR "Stein Leventhal syndrome"[tiab] OR "sclerocystic ovary syndrome"[tiab] OR "sclerocystic ovaries"[tiab] OR "sclerocystic ovarian degeneration"[tiab]
#2 Intervention	"Aerobic Exercise"[Mesh] OR "Exercise"[Mesh] OR "Exercise Therapy"[Mesh] OR "High-Intensity Interval Training"[Mesh] OR "Running"[Mesh] OR "Walking"[Mesh] OR "Bicycling"[Mesh] OR "Swimming"[Mesh] OR "Yoga"[Mesh] OR "Tai Ji"[Mesh] OR "aerobic exercise"[tiab] OR "aerobic exercises"[tiab] OR "aerobic training"[tiab] OR "endurance exercise"[tiab] OR "endurance training"[tiab] OR "exercise training"[tiab] OR "physical exercise"[tiab] OR "physical activity"[tiab] OR "exercise therapy"[tiab] OR running[tiab] OR jogging[tiab] OR walking[tiab] OR cycling[tiab] OR bicycling[tiab] OR swimming[tiab] OR "interval training"[tiab] OR "high-intensity interval training"[tiab] OR "high intensity interval training"[tiab] OR HIIT[tiab] OR "moderate-intensity continuous training"[tiab] OR "moderate intensity continuous training"[tiab] OR MICT[tiab] OR "continuous training"[tiab] OR yoga[tiab] OR "tai chi"[tiab] OR "tai ji"[tiab] OR dance[tiab]
**#3 Final**	**#1 AND #2**

Note. No outcome terms, publication-year limits, or sample-size limits were applied. The search was last run in July 2026.

Supplementary Table S4. Search strategy for Embase (Embase.com).

Search line	Search strategy
#1 Population (Emtree)	'polycystic ovary syndrome'/exp
#2 Population (free text)	'polyendocrine metabolic ovarian syndrome':ti,ab,kw OR PMOS:ti,ab,kw OR 'polycystic ovary syndrome':ti,ab,kw OR 'polycystic ovarian syndrome':ti,ab,kw OR PCOS:ti,ab,kw OR 'Stein-Leventhal syndrome':ti,ab,kw OR 'Stein Leventhal syndrome':ti,ab,kw OR 'sclerocystic ovary syndrome':ti,ab,kw OR 'sclerocystic ovaries':ti,ab,kw OR 'sclerocystic ovarian degeneration':ti,ab,kw
#3 Population set	#1 OR #2
#4 Intervention (Emtree)	'aerobic exercise'/exp OR 'exercise'/exp OR 'exercise therapy'/exp OR 'high intensity interval training'/exp OR 'running'/exp OR 'walking'/exp OR 'cycling'/exp OR 'swimming'/exp OR 'yoga'/exp OR 'tai chi'/exp
#5 Intervention (free text)	'aerobic exercise':ti,ab,kw OR 'aerobic exercises':ti,ab,kw OR 'aerobic training':ti,ab,kw OR 'endurance exercise':ti,ab,kw OR 'endurance training':ti,ab,kw OR 'exercise training':ti,ab,kw OR 'physical exercise':ti,ab,kw OR 'physical activity':ti,ab,kw OR 'exercise therapy':ti,ab,kw OR running:ti,ab,kw OR jogging:ti,ab,kw OR walking:ti,ab,kw OR cycling:ti,ab,kw OR bicycling:ti,ab,kw OR swimming:ti,ab,kw OR 'interval training':ti,ab,kw OR 'high-intensity interval training':ti,ab,kw OR 'high intensity interval training':ti,ab,kw OR HIIT:ti,ab,kw OR 'moderate-intensity continuous training':ti,ab,kw OR 'moderate intensity continuous training':ti,ab,kw OR MICT:ti,ab,kw OR 'continuous training':ti,ab,kw OR yoga:ti,ab,kw OR 'tai chi':ti,ab,kw OR 'tai ji':ti,ab,kw OR dance:ti,ab,kw
#6 Intervention set	#4 OR #5
**#7 Final**	**#3 AND #6**

Note. No outcome terms, publication-year limits, or sample-size limits were applied. The search was last run in July 2026.

Supplementary Table S5. Search strategy for Web of Science Core Collection.

Search line	Search strategy
#1 Population	TS=("polyendocrine metabolic ovarian syndrome" OR PMOS OR "polycystic ovary syndrome" OR "polycystic ovarian syndrome" OR PCOS OR "Stein-Leventhal syndrome" OR "Stein Leventhal syndrome" OR "sclerocystic ovary syndrome" OR "sclerocystic ovaries" OR "sclerocystic ovarian degeneration")
#2 Intervention	TS=("aerobic exercise" OR "aerobic exercises" OR "aerobic training" OR "endurance exercise" OR "endurance training" OR "exercise training" OR "physical exercise" OR "physical activity" OR "exercise therapy" OR running OR jogging OR walking OR cycling OR bicycling OR swimming OR "interval training" OR "high-intensity interval training" OR "high intensity interval training" OR HIIT OR "moderate-intensity continuous training" OR "moderate intensity continuous training" OR MICT OR "continuous training" OR yoga OR "tai chi" OR "tai ji" OR dance)
**#3 Final**	**#1 AND #2**

Note. No outcome terms, publication-year limits, or sample-size limits were applied. The search was last run in July 2026.

Supplementary Table S6. Search strategy for Cochrane Library (CENTRAL).

Search line	Search strategy
#1 Population (MeSH)	MeSH descriptor: [Polycystic Ovary Syndrome] explode all trees
#2 Population (free text)	("polyendocrine metabolic ovarian syndrome" OR PMOS OR "polycystic ovary syndrome" OR "polycystic ovarian syndrome" OR PCOS OR "Stein-Leventhal syndrome" OR "Stein Leventhal syndrome" OR "sclerocystic ovary syndrome" OR "sclerocystic ovaries" OR "sclerocystic ovarian degeneration"):ti,ab,kw
#3 Population set	#1 OR #2
#4 Intervention (MeSH)	MeSH descriptor: [Exercise] explode all trees OR MeSH descriptor: [Exercise Therapy] explode all trees OR MeSH descriptor: [High-Intensity Interval Training] explode all trees OR MeSH descriptor: [Running] explode all trees OR MeSH descriptor: [Walking] explode all trees OR MeSH descriptor: [Bicycling] explode all trees OR MeSH descriptor: [Swimming] explode all trees OR MeSH descriptor: [Yoga] explode all trees OR MeSH descriptor: [Tai Ji] explode all trees
#5 Intervention (free text)	("aerobic exercise" OR "aerobic exercises" OR "aerobic training" OR "endurance exercise" OR "endurance training" OR "exercise training" OR "physical exercise" OR "physical activity" OR "exercise therapy" OR running OR jogging OR walking OR cycling OR bicycling OR swimming OR "interval training" OR "high-intensity interval training" OR "high intensity interval training" OR HIIT OR "moderate-intensity continuous training" OR "moderate intensity continuous training" OR MICT OR "continuous training" OR yoga OR "tai chi" OR "tai ji" OR dance):ti,ab,kw
#6 Intervention set	#4 OR #5
**#7 Final**	**#3 AND #6**

Note. No outcome terms, publication-year limits, or sample-size limits were applied. The search was last run in July 2026.

Supplementary Table S7. Search strategy for Scopus.

Search line	Search strategy
#1 Population	TITLE-ABS-KEY("polyendocrine metabolic ovarian syndrome" OR PMOS OR "polycystic ovary syndrome" OR "polycystic ovarian syndrome" OR PCOS OR "Stein-Leventhal syndrome" OR "Stein Leventhal syndrome" OR "sclerocystic ovary syndrome" OR "sclerocystic ovaries" OR "sclerocystic ovarian degeneration")
#2 Intervention	TITLE-ABS-KEY("aerobic exercise" OR "aerobic exercises" OR "aerobic training" OR "endurance exercise" OR "endurance training" OR "exercise training" OR "physical exercise" OR "physical activity" OR "exercise therapy" OR running OR jogging OR walking OR cycling OR bicycling OR swimming OR "interval training" OR "high-intensity interval training" OR "high intensity interval training" OR HIIT OR "moderate-intensity continuous training" OR "moderate intensity continuous training" OR MICT OR "continuous training" OR yoga OR "tai chi" OR "tai ji" OR dance)
**#3 Final**	**#1 AND #2**

Note. No outcome terms, publication-year limits, or sample-size limits were applied. The search was last run in July 2026.

Part 3. Subgroup Analysis Results

Numerical subgroup results are presented in the order of first appearance in the main manuscript: body mass index, total testosterone, free androgen index, sex hormone-binding globulin, follicle-stimulating hormone, and luteinizing hormone. Corresponding graphical summaries and exploratory meta-regressions are presented in Part 4.

Supplementary Table S8. Subgroup analyses of the effect of aerobic exercise on body mass index (BMI).

Subgroup	k (effect estimates)	Hedges' g	95% CI	p_between
**Obesity status**				**0.25**
Without obesity	15	−0.22	−0.36 to −0.08	
With obesity	4	−0.04	−0.45 to 0.36	
**Supervision status**				**0.50**
Supervised	17	−0.20	−0.34 to −0.05	
Unsupervised	2	−0.14	−0.23 to −0.04	
**Exercise intensity**				**0.21**
High	6	−0.11	−0.25 to 0.02	
Moderate	8	−0.25	−0.46 to −0.03	
Low	5	−0.16	−0.31 to −0.02	
**Exercise modality**				**0.14**
Mind-body exercise	4	−0.16	−0.34 to 0.01	
Combined training	5	+0.03	−0.27 to 0.33	
Cycling	3	−0.45	−1.03 to 0.14	
Running	7	−0.08	−0.21 to 0.06	

Note. Values are Hedges' g with 95% confidence intervals. k is the number of effect estimates, not the number of independent trials. p_between is the omnibus test of subgroup differences. Formal comparisons should be interpreted only when the prespecified independent-trial threshold was met; otherwise, subgroup estimates are descriptive. Negative and positive values indicate lower and higher outcome levels, respectively, with exercise than with a non-exercise control; clinical desirability is outcome-specific.

Supplementary Table S9. Subgroup analyses of the effect of aerobic exercise on total testosterone (T).

Subgroup	k (effect estimates)	Hedges' *g*	95% CI	p_between
**Obesity status**				**0.62**
Without obesity	14	−0.45	−0.66 to −0.22	
With obesity	6	−0.39	−0.82 to 0.4	
**Supervision status**				**0.39**
Supervised	17	−0.46	−0.72 to −0.19	
Unsupervised	3	−0.17	−2.58 to 2.25	
**Exercise intensity**				**0.99**
High	10	−0.43	−0.91 to 0.06	
Moderate	7	−0.44	−0.75 to −0.13	
Low	3	−0.43	−1.13 to 0.29	
**Exercise modality**				**0.25**
Mind-body exercise	2	−0.53	−1.58 to 0.52	
Combined training	6	−0.29	−0.53 to −0.06	
Cycling	2	−0.17	−0.18 to −0.16	
Running	10	−0.61	−1.27 to 0.05	

Note. Values are Hedges' g with 95% confidence intervals. k is the number of effect estimates, not the number of independent trials. p_between is the omnibus test of subgroup differences. Formal comparisons should be interpreted only when the prespecified independent-trial threshold was met; otherwise, subgroup estimates are descriptive. Negative and positive values indicate lower and higher outcome levels, respectively, with exercise than with a non-exercise control; clinical desirability is outcome-specific.

Supplementary Table S10. Subgroup analyses of the effect of aerobic exercise on the free androgen index (FAI).

Subgroup	k (effect estimates)	Hedges' *g*	95% CI	p_between
**Obesity status**				**0.95**
Without obesity	8	−0.04	−0.28 to 0.20	
With obesity	4	−0.06	−1.07 to 0.95	
**Supervision status**				**0.53**
Supervised	9	−0.07	−0.39 to 0.26	
Unsupervised	3	0.06	−0.48 to 0.59	
**Exercise intensity**				**0.71**
High	5	0.11	−0.76 to 0.98	
Moderate	5	−0.16	−0.54 to 0.22	
Low	2	0.02	−2.96 to 3.01	
**Exercise modality**				**0.33**
Mind-body exercise	1	−0.16	N/A	
Combined training	4	0.23	−0.01 to 0.47	
Cycling	2	−0.17	−0.18 to 0.84	
Running	5	−0.08	−1.38 to 1.23	

Note. Values are Hedges' g with 95% confidence intervals. k is the number of effect estimates, not the number of independent trials. p_between is the omnibus test of subgroup differences. Formal comparisons should be interpreted only when the prespecified independent-trial threshold was met; otherwise, subgroup estimates are descriptive. Negative and positive values indicate lower and higher outcome levels, respectively, with exercise than with a non-exercise control; clinical desirability is outcome-specific.

Supplementary Table S11. Subgroup analyses of the effect of aerobic exercise on sex hormone-binding globulin (SHBG).

**Subgroup**	k (effect estimates)	**Hedges' *g***	**95% CI**	p_between
**Obesity status**				**0.08**
Without obesity	11	0.29	0.09 to 0.49	
With obesity	5	0.07	−0.15 to 0.28	
**Supervision status**				**0.58**
Supervised	13	0.24	0.02 to 0.47	
Unsupervised	3	0.17	−0.36 to 0.70	
**Exercise intensity**				**0.21**
High	7	0.15	−0.18 to 0.29	
Moderate	7	0.32	−0.01 to 0.65	
Low	2	0.35	−0.32 to 1.02	
**Exercise modality**				**0.07**
Mind-body exercise	1	0.38	N/A	
Combined training	7	0.17	−0.10 to 0.44	
Cycling	2	0.47	0.40 to 0.55	
Running	6	0.00	−0.26 to 0.25	

Note. Values are Hedges' g with 95% confidence intervals. k is the number of effect estimates, not the number of independent trials. p_between is the omnibus test of subgroup differences. Formal comparisons should be interpreted only when the prespecified independent-trial threshold was met; otherwise, subgroup estimates are descriptive. Negative and positive values indicate lower and higher outcome levels, respectively, with exercise than with a non-exercise control; clinical desirability is outcome-specific.

Supplementary Table S12. Subgroup analyses of the effect of aerobic exercise on follicle-stimulating hormone (FSH).

Subgroup	k (effect estimates)	Hedges' *g*	95% CI	p_between
**Obesity status**				**0.83**
Without obesity	11	0.37	−0.10 to 0.84	
With obesity	2	0.45	−2.72 to 3.36	
**Exercise intensity**				**0.56**
High	2	0.16	−0.09 to 0.41	
Moderate	8	0.53	−0.14 to 1.20	
Low	3	0.15	−0.18 to 0.49	
**Exercise modality**				**0.81**
Mind-body exercise	2	0.12	−0.98 to 1.23	
Combined training	5	0.80	−0.98 to 2.58	
Cycling	3	0.16	−0.23 to 0.55	
Running	3	0.40	−3.01 to 3.87	

Note. Values are Hedges' g with 95% confidence intervals. k is the number of effect estimates, not the number of independent trials. p_between is the omnibus test of subgroup differences. Formal comparisons should be interpreted only when the prespecified independent-trial threshold was met; otherwise, subgroup estimates are descriptive. Negative and positive values indicate lower and higher outcome levels, respectively, with exercise than with a non-exercise control; clinical desirability is outcome-specific.

Supplementary Table S13. Subgroup analyses of the effect of aerobic exercise on luteinizing hormone (LH).

Subgroup	k (effect estimates)	Hedges' *g*	95% CI	p_between
**Obesity status**				**0.48**
Without obesity	11	−0.25	−0.59 to 0.09	
With obesity	2	−0.90	−9.44 to 7.65	
**Exercise intensity**				**0.82**
High	2	−0.25	−2.25 to 1.75	
Moderate	8	−0.42	−0.94 to 0.10	
Low	3	−0.21	−2.41 to 1.99	
**Exercise modality**				**0.79**
Mind-body exercise	2	−0.38	−7.81 to 7.04	
Combined training	5	−0.35	−1.24 to 0.53	
Cycling	3	−0.06	−0.14 to 0.10	
Running	3	−0.81	−9.39 to 7.77	

Note. Values are Hedges' g with 95% confidence intervals. k is the number of effect estimates, not the number of independent trials. p_between is the omnibus test of subgroup differences. Formal comparisons should be interpreted only when the prespecified independent-trial threshold was met; otherwise, subgroup estimates are descriptive. Negative and positive values indicate lower and higher outcome levels, respectively, with exercise than with a non-exercise control; clinical desirability is outcome-specific.

Part 4. Graphical Subgroup, Meta-Regression, and Dose-Response Results

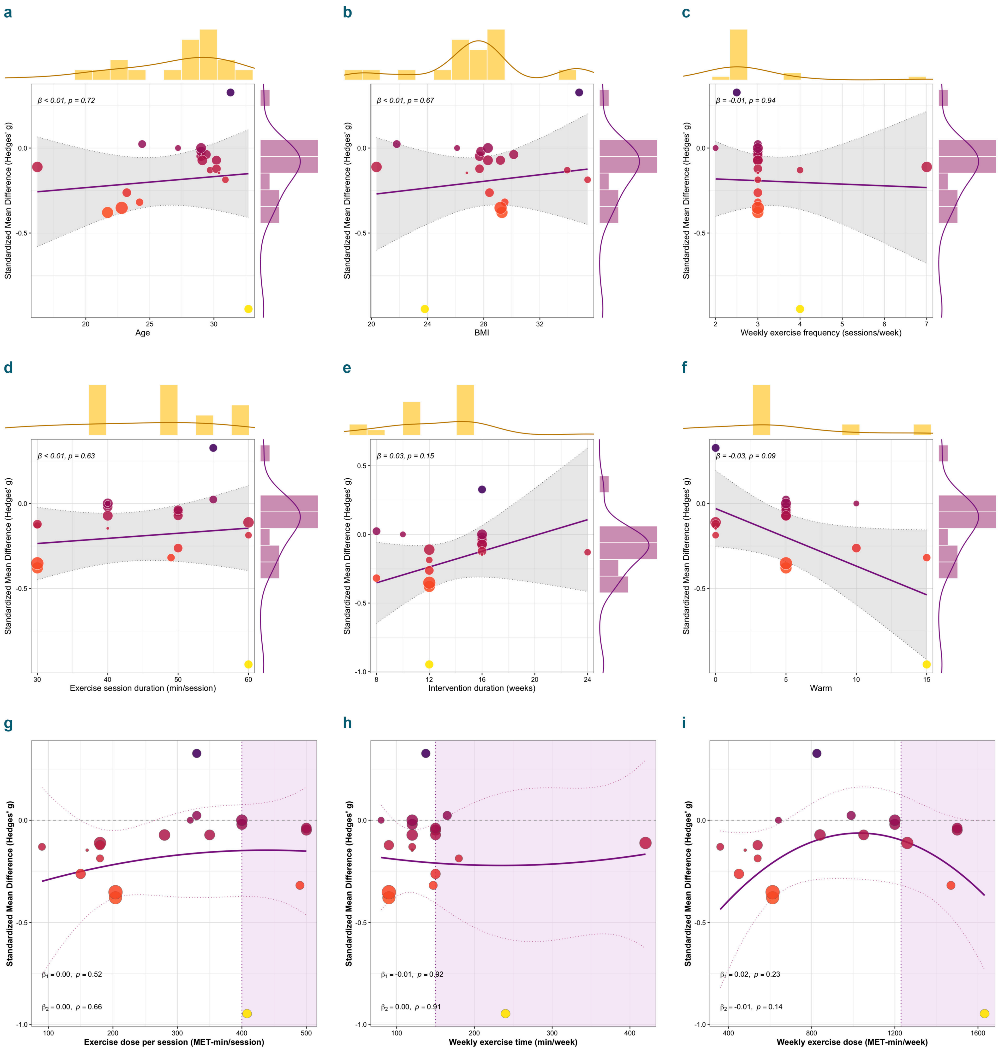

Supplementary Figure S1. BMI meta-regression and dose-response analyses.

Note. Panels (a-f) show exploratory meta-regressions by mean age, baseline BMI, weekly exercise frequency, exercise-session duration, intervention duration, and warm-up duration, respectively. Panels (g-i) show quadratic dose-response models for exercise dose per session, weekly exercise time, and weekly exercise dose. Bubble size reflects study precision; fitted lines or curves are accompanied by 95% confidence intervals. The displayed beta coefficients and two-sided p values are model-based exploratory estimates.

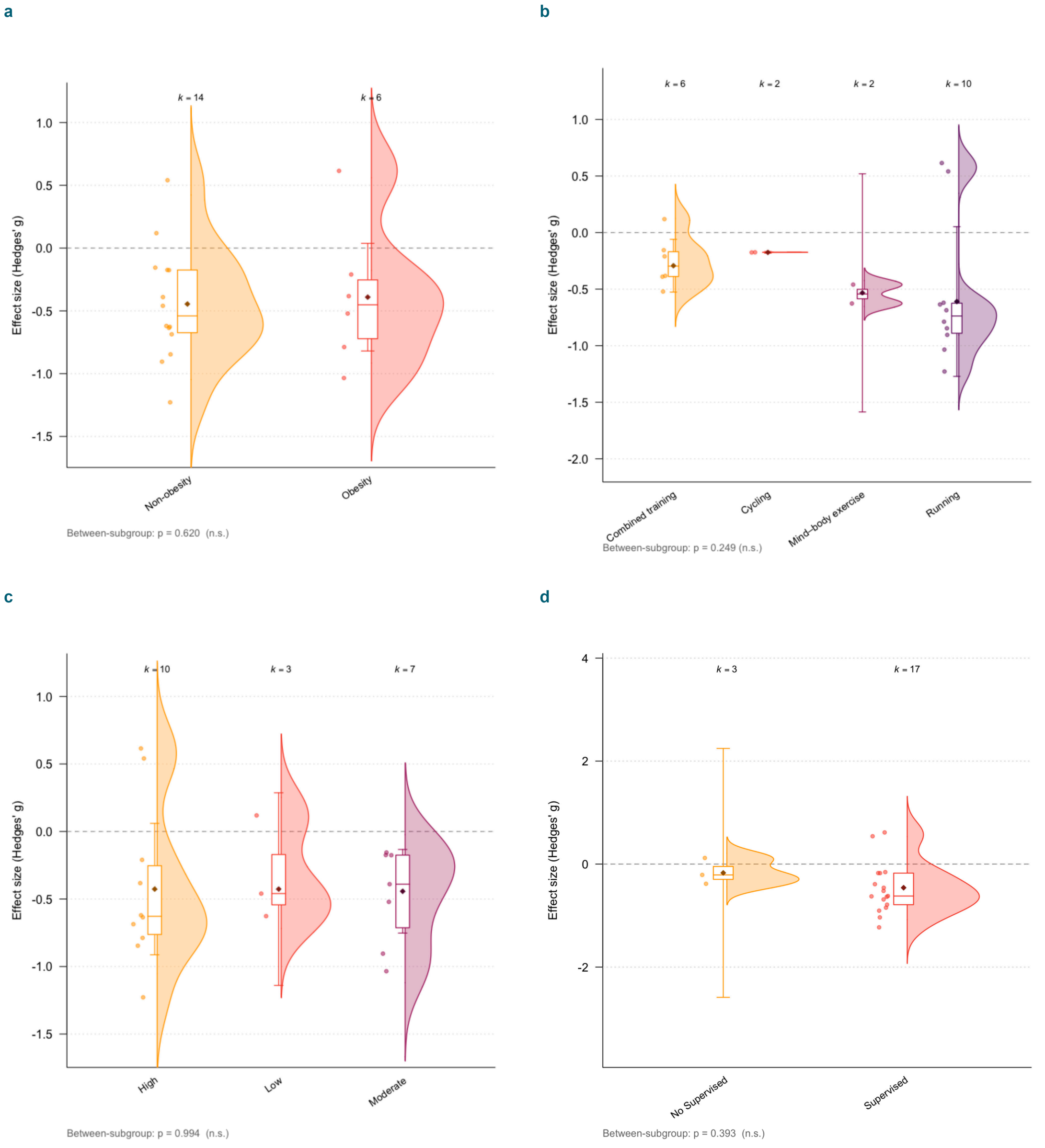

Supplementary Figure S2. Subgroup analyses for total testosterone.

Note. Panels (a-d) show obesity status, exercise modality, exercise intensity, and supervision status, respectively. Points are individual effect estimates; half-violin distributions and box plots summarize within-subgroup distributions. Diamonds and error bars show subgroup pooled estimates and 95% confidence intervals. Between-subgroup tests are exploratory; k denotes the number of effect estimates.

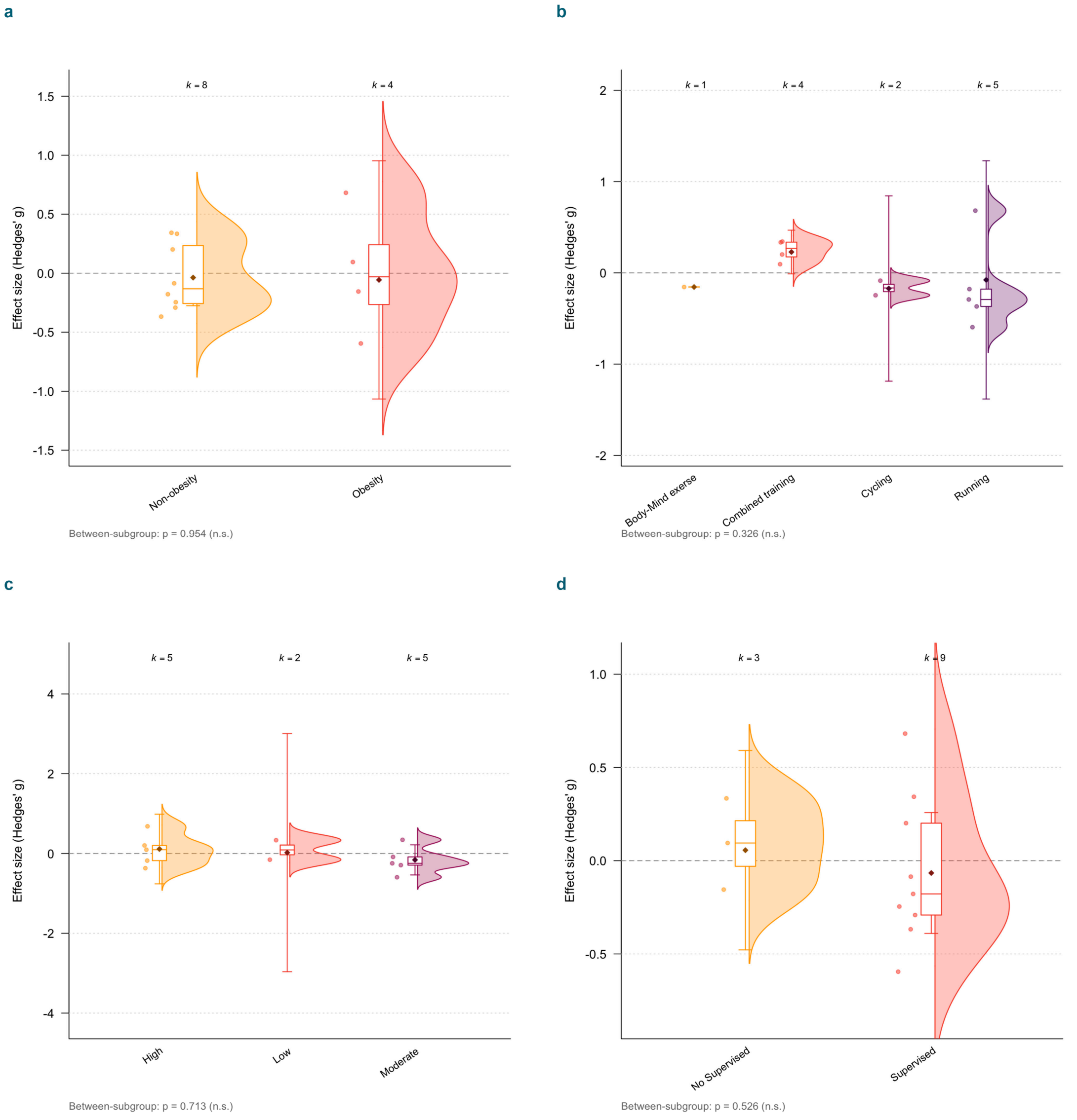

Supplementary Figure S3. Subgroup analyses for the free androgen index.

Note. Panels (a-d) show obesity status, exercise modality, exercise intensity, and supervision status, respectively. Points are individual effect estimates; half-violin distributions and box plots summarize within-subgroup distributions. Diamonds and error bars show subgroup pooled estimates and 95% confidence intervals. Between-subgroup tests are exploratory; k denotes the number of effect estimates.

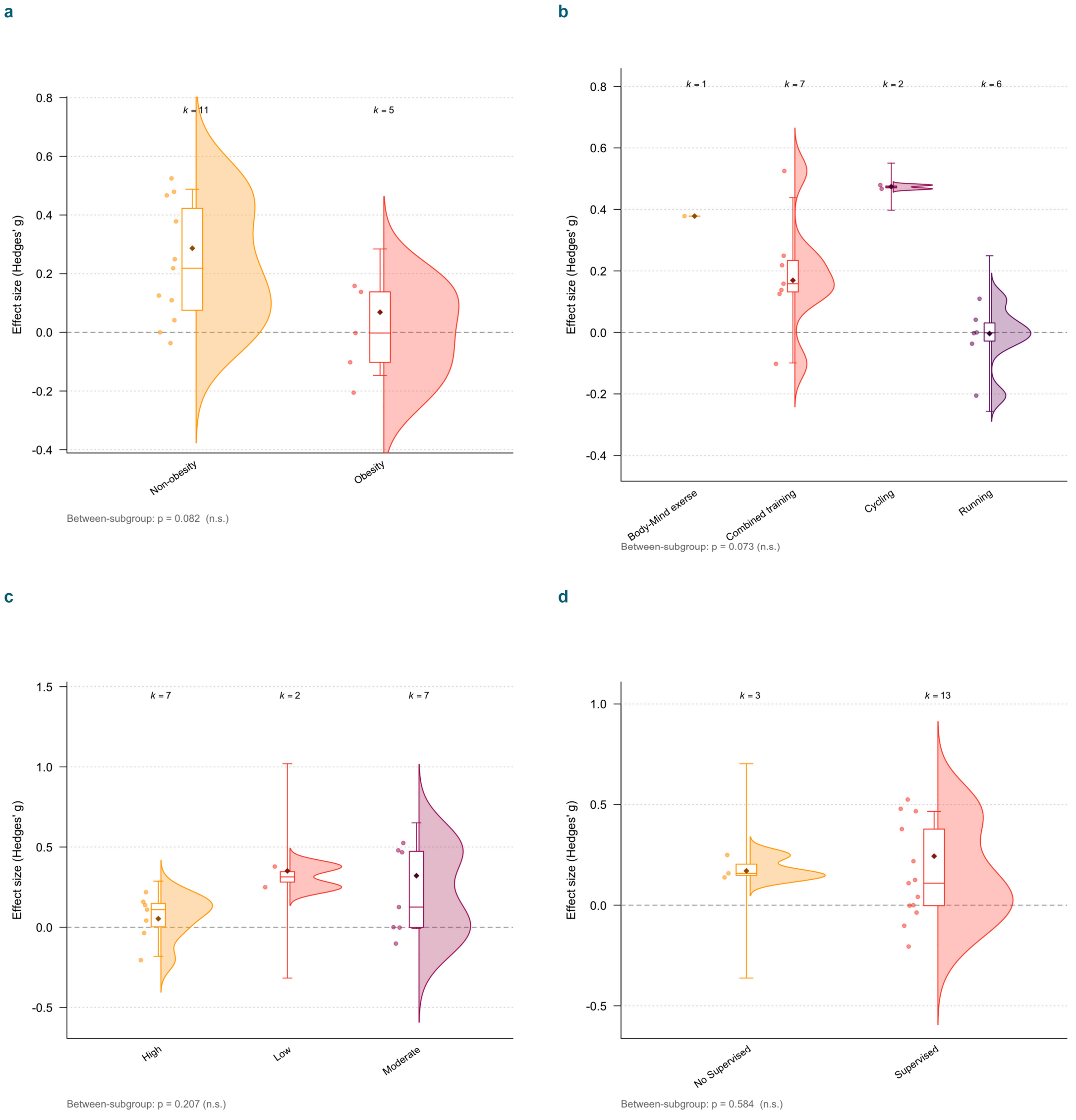

Supplementary Figure S4. Subgroup analyses for sex hormone-binding globulin.

Note. Panels (a-d) show obesity status, exercise modality, exercise intensity, and supervision status, respectively. Points are individual effect estimates; half-violin distributions and box plots summarize within-subgroup distributions. Diamonds and error bars show subgroup pooled estimates and 95% confidence intervals. Positive values indicate higher sex hormone-binding globulin levels with exercise. Between-subgroup tests are exploratory; k denotes the number of effect estimates.

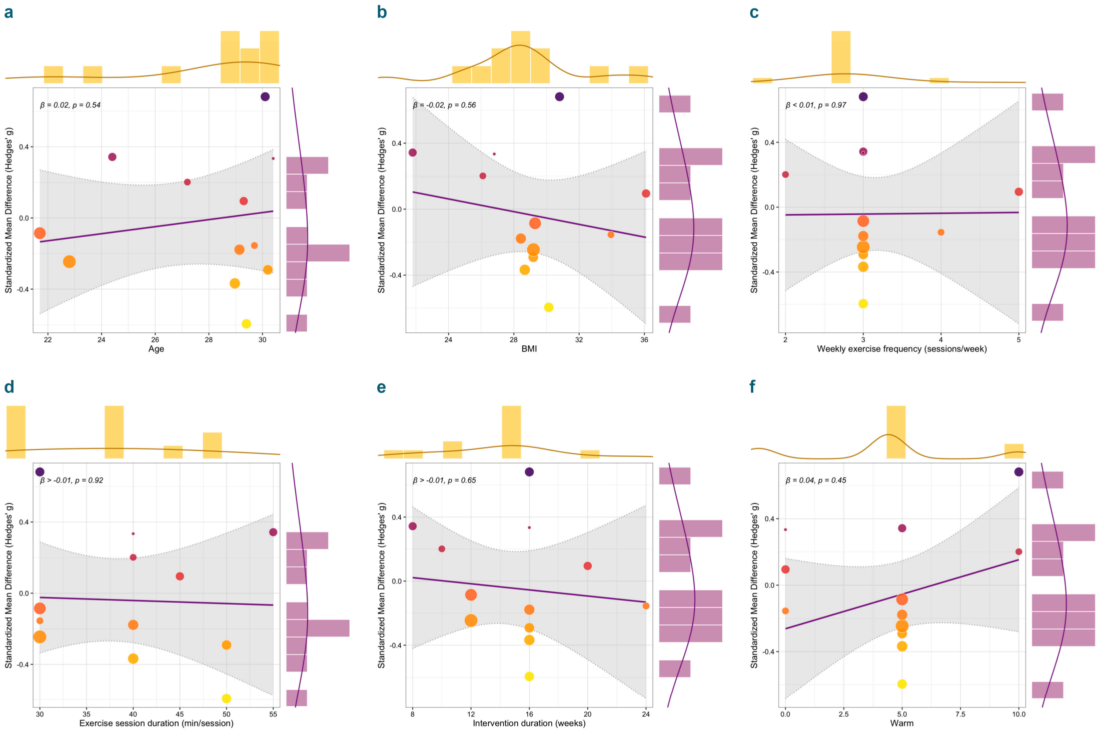

Supplementary Figure S5. Meta-regression analyses for the free androgen index.

Note. Panels (a-f) show exploratory bubble meta-regressions by mean age, baseline BMI, weekly exercise frequency, exercise-session duration, intervention duration, and warm-up duration, respectively. Bubble size reflects study precision; fitted slopes are accompanied by 95% confidence intervals. The displayed beta coefficients and two-sided p values were not adjusted for multiple testing.

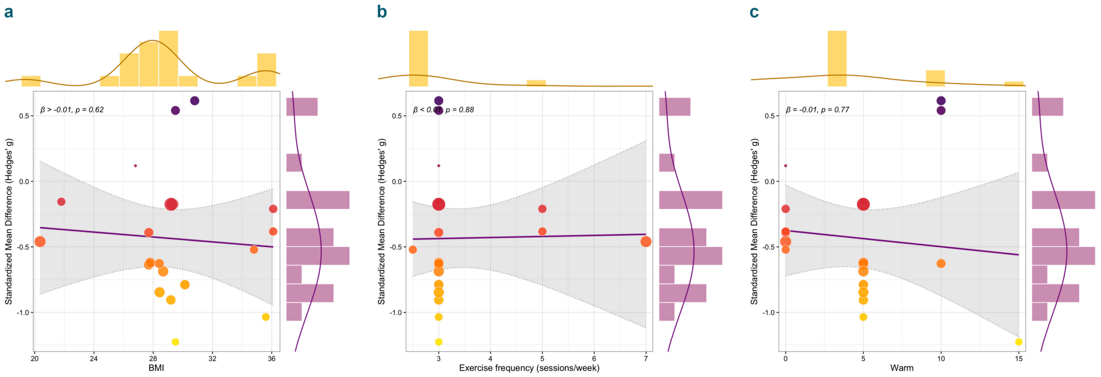

Supplementary Figure S6. Additional meta-regression analyses for total testosterone.

Note. Panels (a-c) show exploratory bubble meta-regressions by baseline BMI, weekly exercise frequency, and warm-up duration, respectively. Moderators presented in the main figures are not duplicated. Bubble size reflects study precision; fitted slopes are accompanied by 95% confidence intervals. The displayed beta coefficients and two-sided p values were not adjusted for multiple testing.

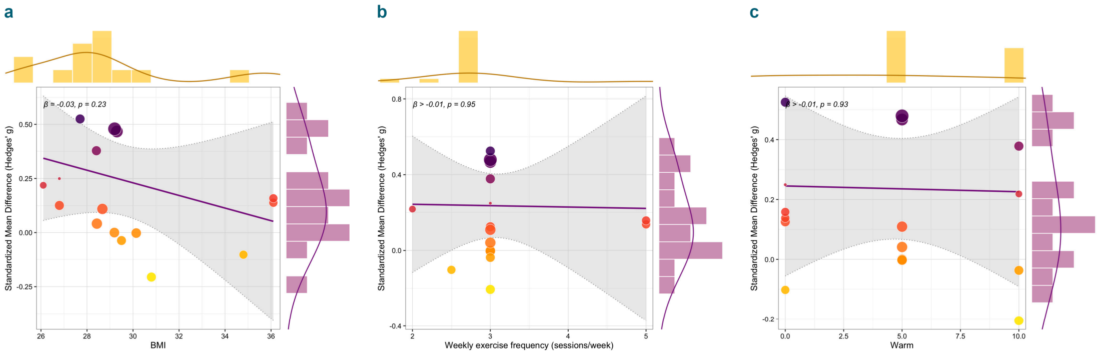

Supplementary Figure S7. Additional meta-regression analyses for sex hormone-binding globulin.

Note. Panels (a-c) show exploratory bubble meta-regressions by baseline BMI, weekly exercise frequency, and warm-up duration, respectively. Moderators presented in the main figures are not duplicated. Bubble size reflects study precision; fitted slopes are accompanied by 95% confidence intervals. The displayed beta coefficients and two-sided p values were not adjusted for multiple testing.

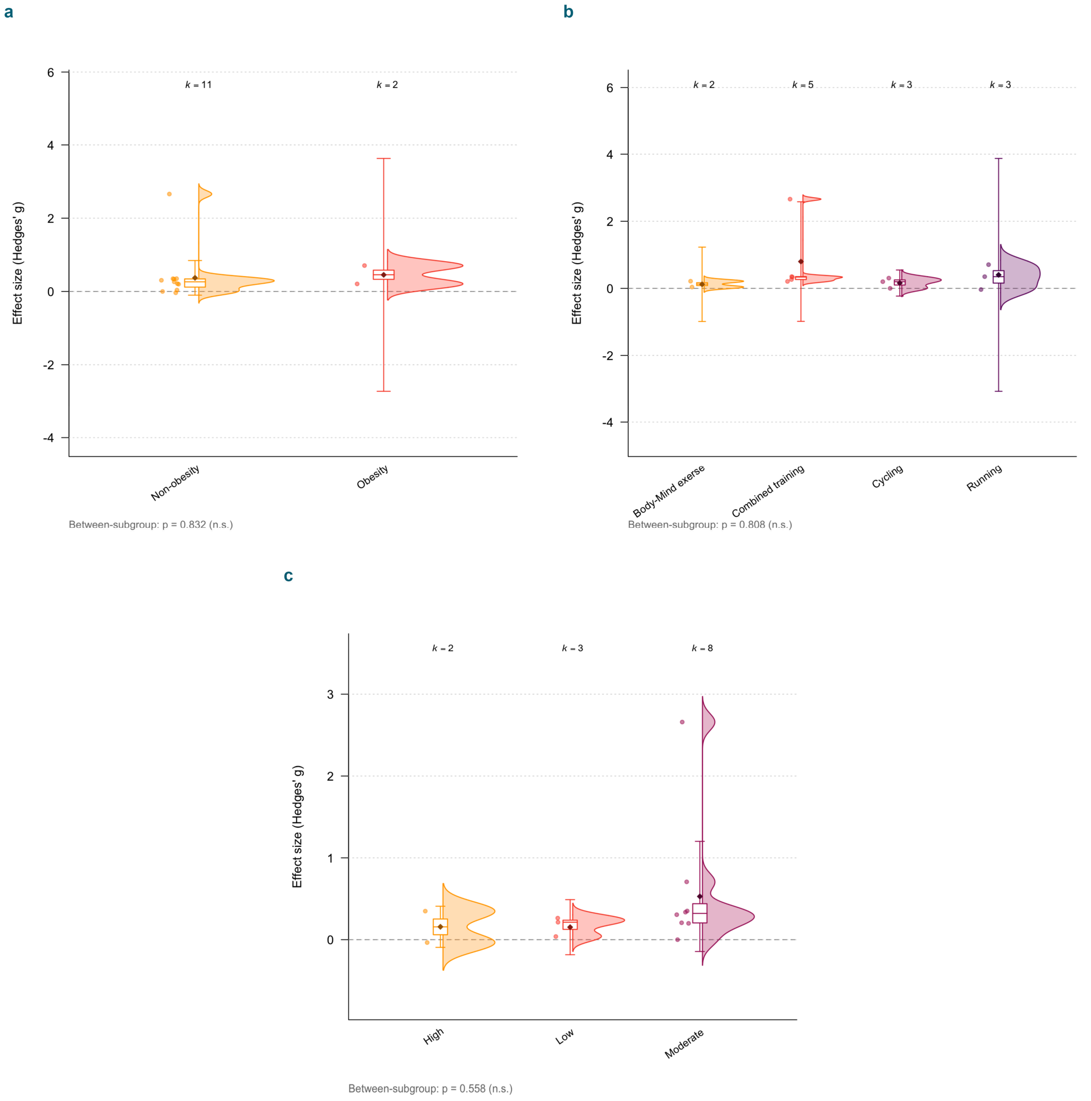

Supplementary Figure S8. Subgroup analyses for follicle-stimulating hormone.

Note. Panels (a-c) show obesity status, exercise modality, and exercise intensity, respectively. Points are individual effect estimates; half-violin distributions and box plots summarize within-subgroup distributions. Diamonds and error bars show subgroup pooled estimates and 95% confidence intervals. Between-subgroup tests are exploratory; k denotes the number of effect estimates.

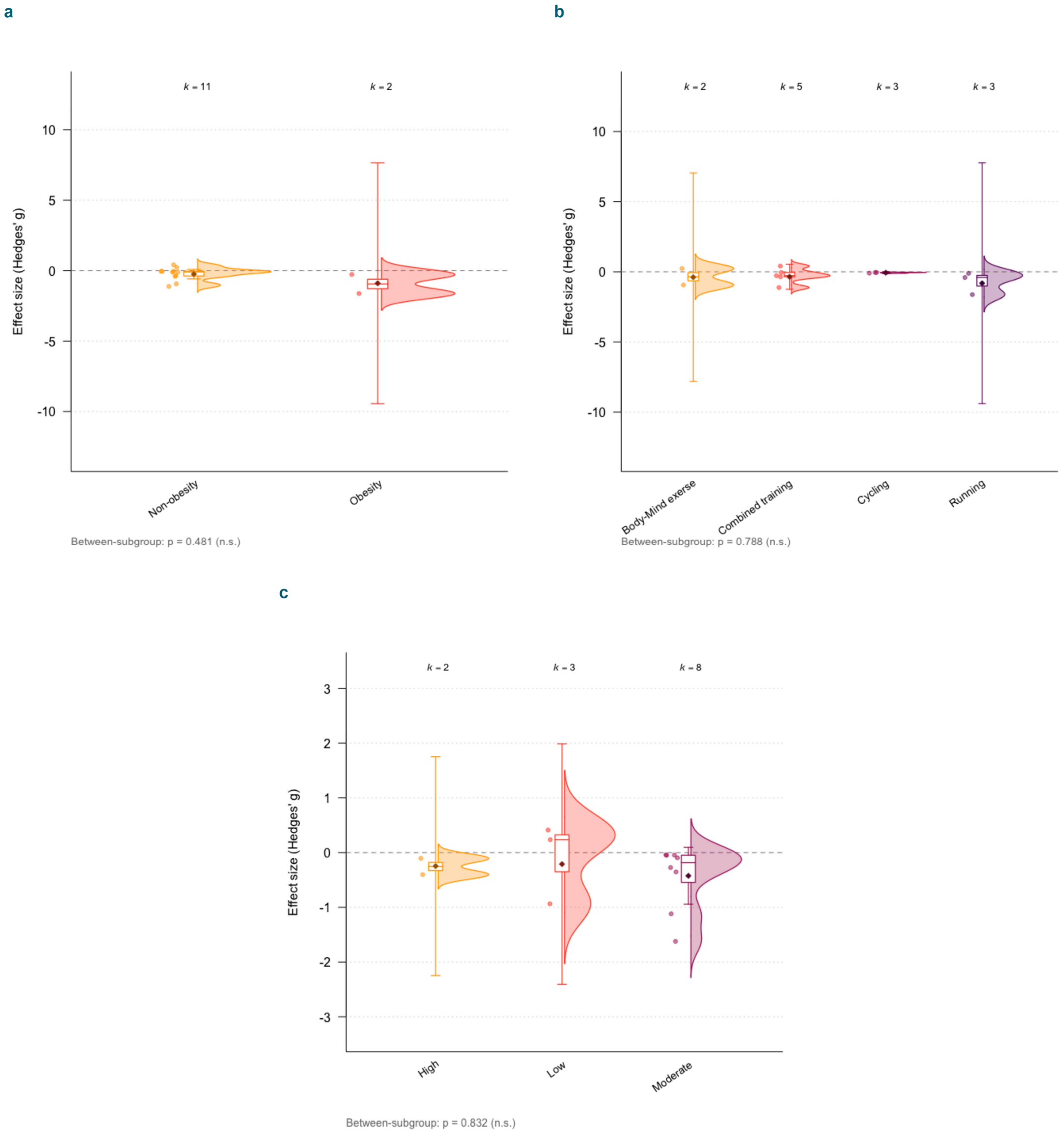

Supplementary Figure S9. Subgroup analyses for luteinizing hormone.

Note. Panels (a-c) show obesity status, exercise modality, and exercise intensity, respectively. Points are individual effect estimates; half-violin distributions and box plots summarize within-subgroup distributions. Diamonds and error bars show subgroup pooled estimates and 95% confidence intervals. Between-subgroup tests are exploratory; k denotes the number of effect estimates.

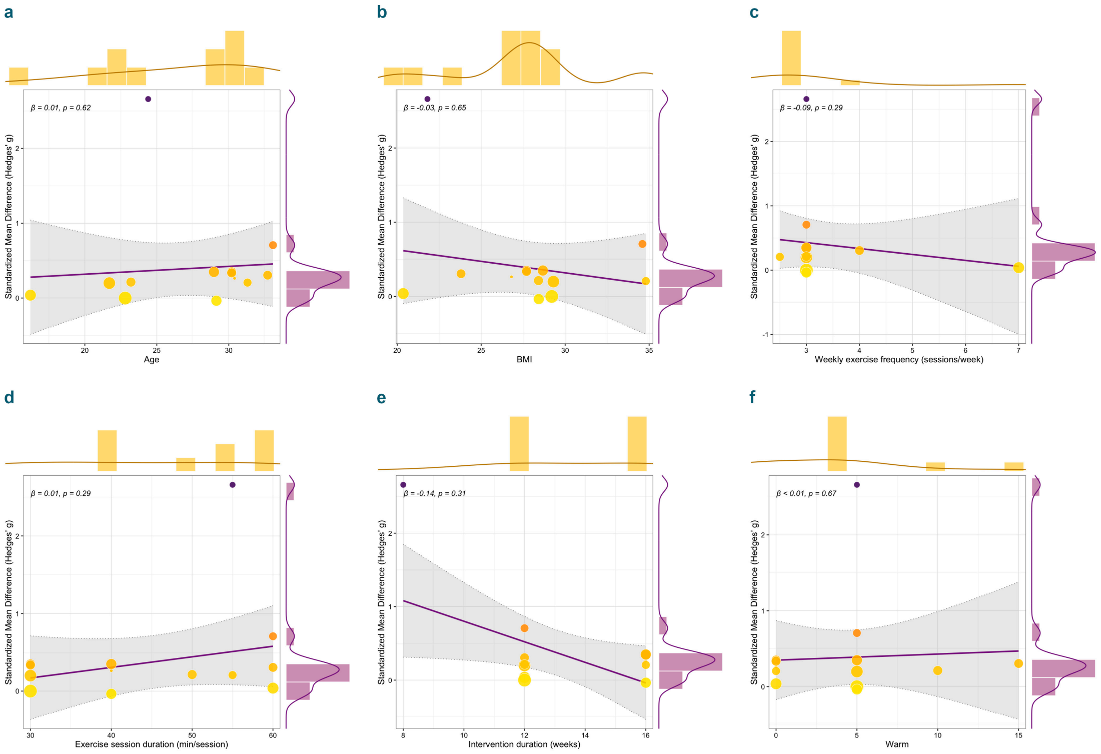

Supplementary Figure S10. Meta-regression analyses for follicle-stimulating hormone.

Note. Panels (a-f) show exploratory bubble meta-regressions by mean age, baseline BMI, weekly exercise frequency, exercise-session duration, intervention duration, and warm-up duration, respectively. Bubble size reflects study precision; fitted slopes are accompanied by 95% confidence intervals. The displayed beta coefficients and two-sided p values were not adjusted for multiple testing.

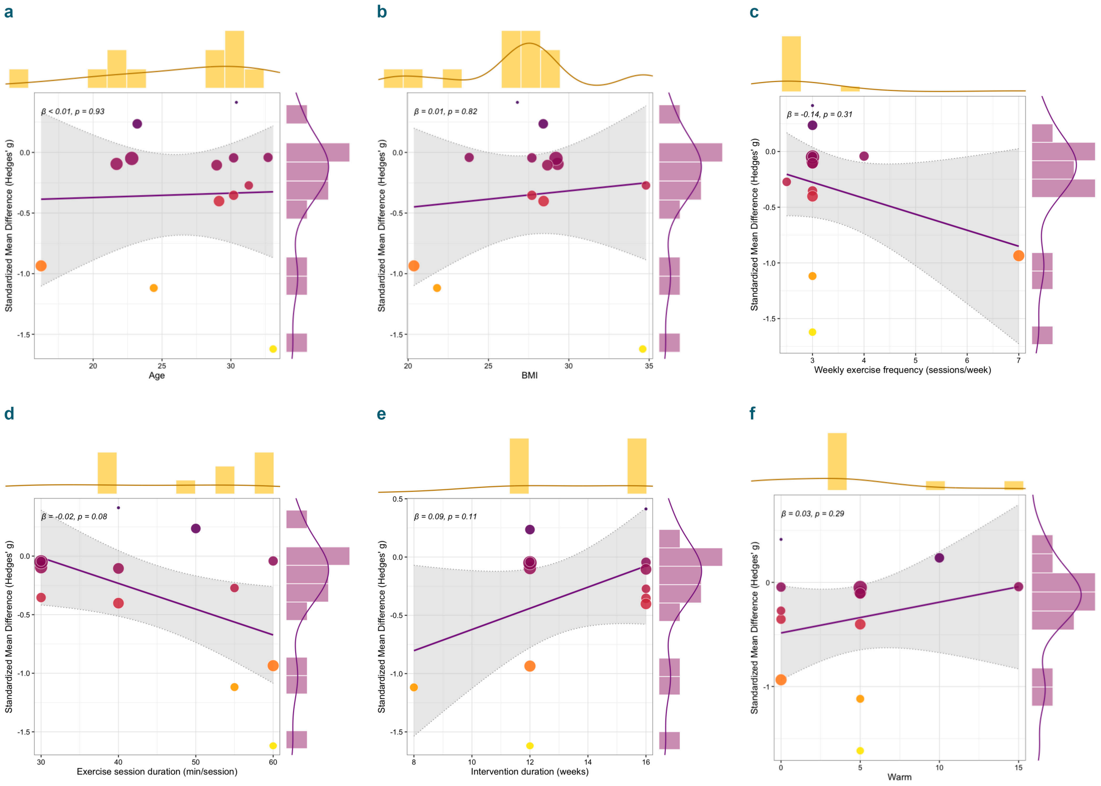

Supplementary Figure S11. Meta-regression analyses for luteinizing hormone.

Note. Panels (a-f) show exploratory bubble meta-regressions by mean age, baseline BMI, weekly exercise frequency, exercise-session duration, intervention duration, and warm-up duration, respectively. Bubble size reflects study precision; fitted slopes are accompanied by 95% confidence intervals. The displayed beta coefficients and two-sided p values were not adjusted for multiple testing.

Part 5. Publication Bias Assessment

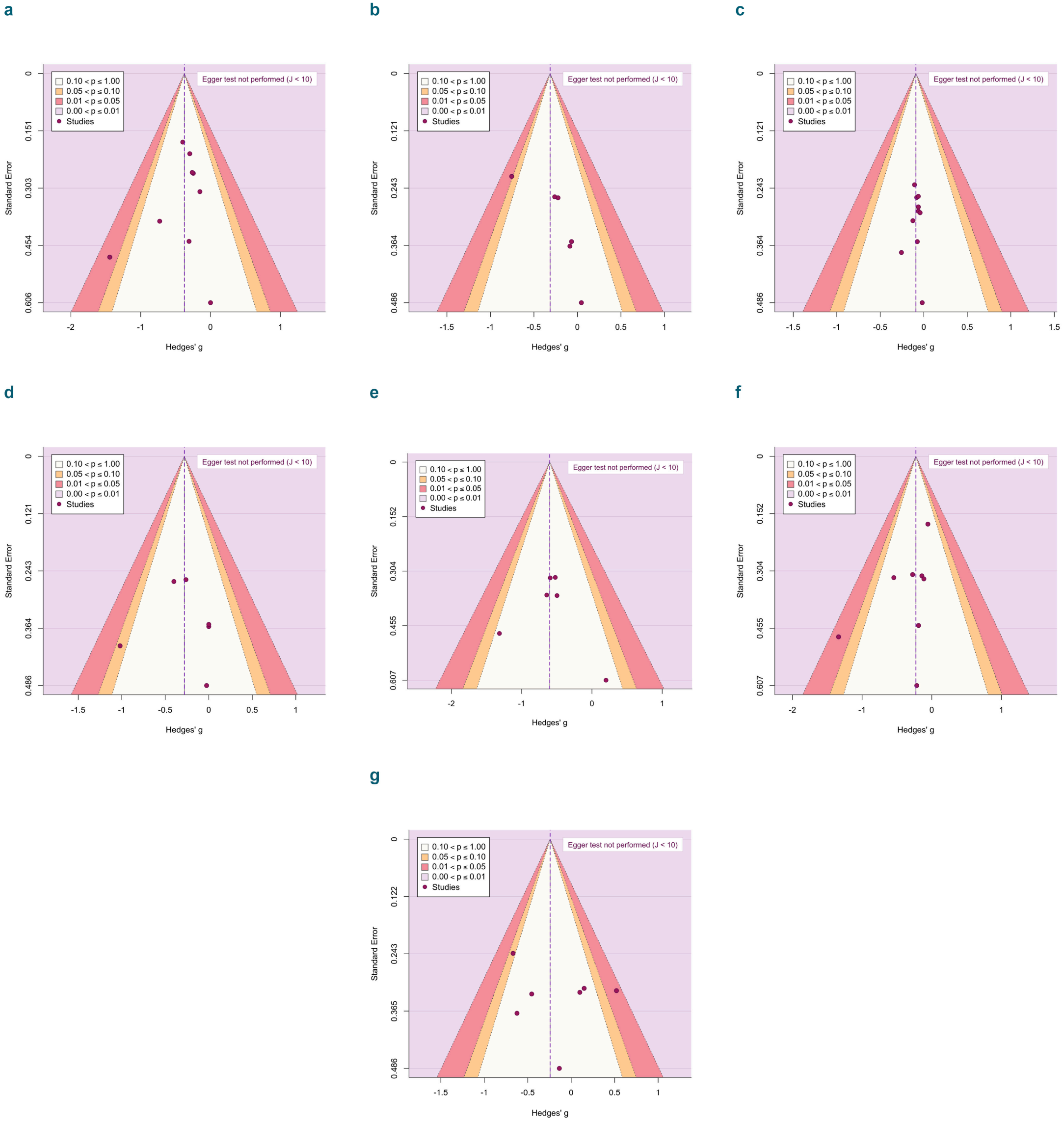

Supplementary Figure S12. Additional contour-enhanced funnel plots.

Note. Panels (a-g) show waist-to-hip ratio, waist circumference, body weight, body fat percentage, free testosterone, dehydroepiandrosterone sulfate, and anti-Müllerian hormone, respectively. Points represent individual effect estimates; vertical dashed lines mark pooled Hedges' g estimates, and contours show two-sided significance regions. Formal asymmetry testing was not performed for outcomes with fewer than 10 independent studies.

Part 6. Sensitivity Analyses

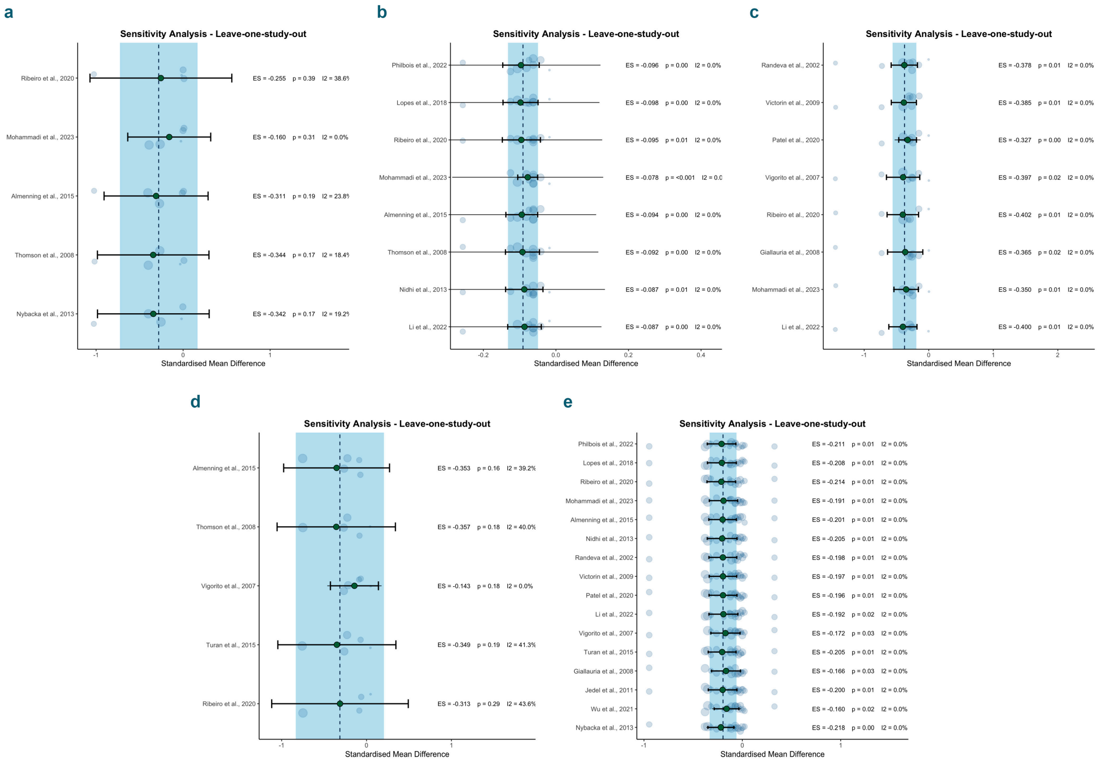

Supplementary Figure S13. Leave-one-study-out sensitivity analyses for anthropometric outcomes.

Note. Panels (a-e) show body fat percentage, body weight, waist-to-hip ratio, waist circumference, and BMI, respectively. Points and horizontal bars show pooled Hedges' g estimates and 95% confidence intervals after omission of the named study. The vertical dashed line marks the full-sample estimate and the shaded band its 95% confidence interval.

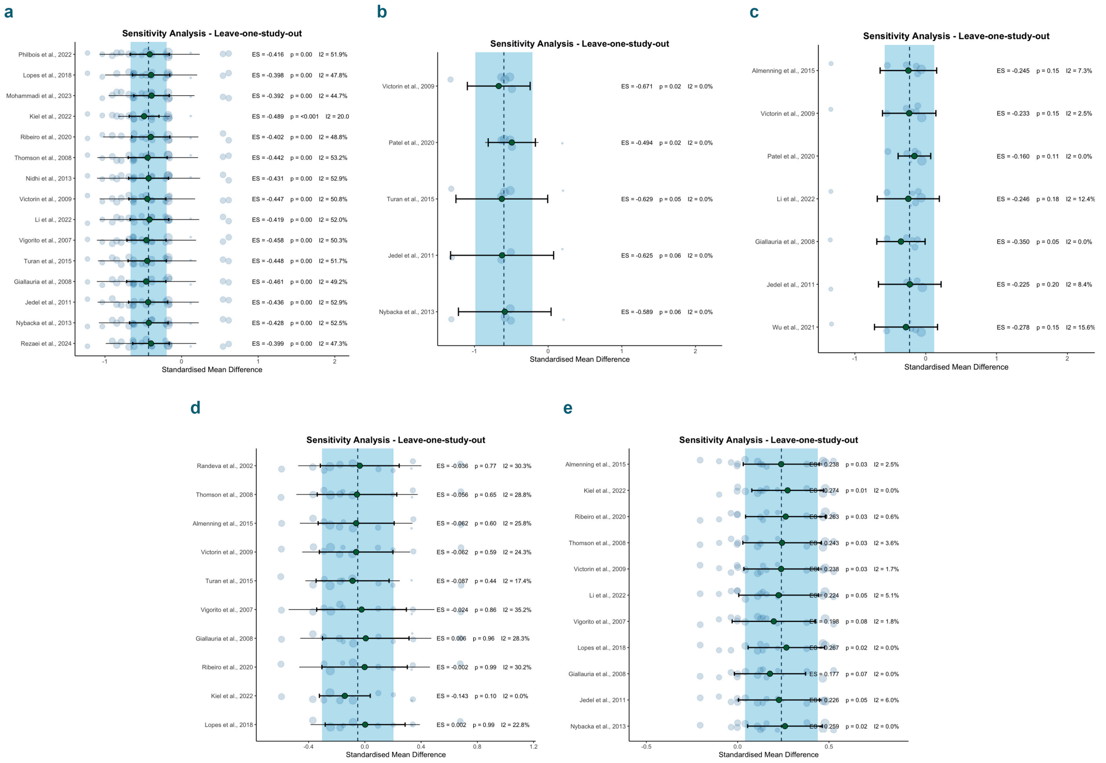

Supplementary Figure S14. Leave-one-study-out sensitivity analyses for androgen-related outcomes.

Note. Panels (a-e) show total testosterone, free testosterone, dehydroepiandrosterone sulfate, free androgen index, and sex hormone-binding globulin, respectively. Points and horizontal bars show pooled Hedges' g estimates and 95% confidence intervals after omission of the named study. The vertical dashed line marks the full-sample estimate and the shaded band its 95% confidence interval.

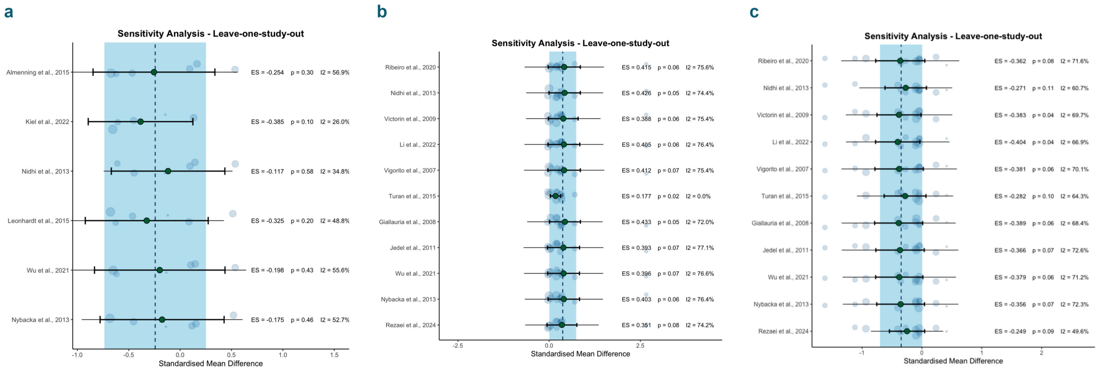

Supplementary Figure S15. Leave-one-study-out sensitivity analyses for reproductive endocrine outcomes.

Note. Panels (a-c) show anti-Müllerian hormone, follicle-stimulating hormone, and luteinizing hormone, respectively. Points and horizontal bars show pooled Hedges' g estimates and 95% confidence intervals after omission of the named study. The vertical dashed line marks the full-sample estimate and the shaded band its 95% confidence interval.

Part 7. GRADE Certainty of Evidence

Supplementary Table S14. GRADE assessment of the certainty of evidence for all outcomes.

Outcome	Evidence base	GRADE domains					Effect estimate	Certainty of evidence
		Risk of bias	Inconsistency	Indirectness	Imprecision	Publication bias		
**Anthropometric outcomes**								
**Body weight**	422 participants<br>8 studies; k=11	Serious [1]	Not serious	Not serious	Serious [2]	Not assessed (<10 independent studies)	Hedges' g -0.09 (95% CI -0.13 to -0.05)<br>95% PI -0.29 to 0.11; I²=0%	⊕⊕○○<br>**LOW**
**Body mass index**	802 participants<br>16 studies; k=19	Serious [1]	Not serious	Not serious	Serious [2]	Not detected (Egger p=0.240)	Hedges' g -0.20 (95% CI -0.33 to -0.06)<br>95% PI -0.34 to -0.05; I²=0%	⊕⊕○○<br>**LOW**
**Waist-to-hip ratio**	425 participants<br>8 studies; k=9	Serious [1]	Not serious	Not serious	Serious [2]	Not assessed (<10 independent studies)	Hedges' g -0.36 (95% CI -0.56 to -0.19)<br>95% PI -0.57 to -0.18; I²=0%	⊕⊕○○<br>**LOW**
**Waist circumference**	256 participants<br>5 studies; k=6	Serious [1]	Not serious	Not serious	Very serious [2a]	Not assessed (<10 independent studies)	Hedges' g -0.31 (95% CI -0.83 to 0.21)<br>95% PI -0.86 to 0.23; I²=34.7%	⊕○○○<br>**VERY LOW**
**Body fat**	195 participants<br>5 studies; k=6	Serious [1]	Not serious	Not serious	Serious [2]	Not assessed (<10 independent studies)	Hedges' g -0.28 (95% CI -0.73 to 0.17)<br>95% PI -0.58 to 0.01; I²=0%	⊕⊕○○<br>**LOW**
**Androgenic outcomes**								
**Total testosterone**	824 participants<br>15 studies; k=20	Serious [1]	Not serious	Not serious	Serious [2]	Not detected (Egger p=0.874)	Hedges' g -0.43 (95% CI -0.67 to -0.20)<br>95% PI -1.04 to 0.18; I²=49.0%	⊕⊕○○<br>**LOW**
**Free testosterone**	139 participants<br>5 studies; k=6	Serious [1]	Not serious	Not serious	Serious [2]	Not assessed (<10 independent studies)	Hedges' g -0.60 (95% CI -0.99 to -0.21)<br>95% PI -0.94 to -0.26; I²=0%	⊕⊕○○<br>**LOW**
**Free androgen index**	524 participants<br>10 studies; k=12	Serious [1]	Not serious	Not serious	Serious [2]	Not serious [4] (Egger p=0.035)	Hedges' g -0.05 (95% CI -0.31 to 0.20)<br>95% PI -0.43 to 0.33; I²=22.7%	⊕⊕○○<br>**LOW**
**Dehydroepiandrosterone sulfate**	302 participants<br>7 studies; k=8	Serious [1]	Not serious	Not serious	Serious [2]	Not assessed (<10 independent studies)	Hedges' g -0.23 (95% CI -0.58 to 0.12)<br>95% PI -0.45 to 0.00; I²=0%	⊕⊕○○<br>**LOW**
**Sex hormone-binding globulin**	609 participants<br>11 studies; k=16	Serious [1]	Not serious	Not serious	Serious [2]	Not detected (Egger p=0.153)	Hedges' g +0.24 (95% CI 0.04 to 0.44)<br>95% PI 0.07 to 0.41; I²=0.4%	⊕⊕○○<br>**LOW**
**Reproductive endocrine outcomes**								
**Anti-Müllerian hormone**	261 participants<br>6 studies; k=7	Serious [1]	Not serious	Not serious	Very serious [2a]	Not assessed (<10 independent studies)	Hedges' g -0.24 (95% CI -0.74 to 0.25)<br>95% PI -0.97 to 0.48; I²=48.8%	⊕○○○<br>**VERY LOW**

Outcome	Evidence base	GRADE domains					Effect estimate	Certainty of evidence
		Risk of bias	Inconsistency	Indirectness	Imprecision	Publication bias		
**Follicle-stimulating hormone**	600 participants<br>11 studies; k=13	Serious [1]	Serious [3]	Not serious	Serious [2]	Not detected (Egger p=0.170)	Hedges' g +0.37 (95% CI 0.01 to 0.74)<br>95% PI -0.56 to 1.30; I^2=70.4%	⨁○○○<br>**VERY LOW**
**Luteinizing hormone**	600 participants<br>11 studies; k=13	Serious [1]	Serious [3]	Not serious	Serious [2]	Not detected (Egger p=0.752)	Hedges' g -0.34 (95% CI -0.70 to -0.01)<br>95% PI -1.23 to 0.53; I^2=67.9%	⨁○○○<br>**VERY LOW**

GRADE judgments and explanatory notes

[1] Risk of bias - serious (downgrade one level). Most outcome-specific evidence was contributed by trials with some concerns or high-risk judgments in the available RoB 2 assessments, particularly for the randomization process and selection of the reported result.

[2] Imprecision - serious (downgrade one level). A one-level downgrade was applied when the outcome-specific evidence base was small or fragmented and confidence or prediction intervals retained material uncertainty.

[2a] Imprecision - very serious (downgrade two levels). Waist circumference and anti-Müllerian hormone were supported by small evidence bases with wide confidence and prediction intervals crossing the null; a two-level downgrade was applied.

[3] Inconsistency - serious for follicle-stimulating hormone and luteinizing hormone (downgrade one level). Heterogeneity was substantial (I2=70.4% and 67.9%, respectively), and both prediction intervals crossed the null, indicating that the pooled means may not be reproducible across comparable settings.

[4] Reporting bias - not downgraded. Egger's test indicated possible small-study effects for the free androgen index (p=0.035), but this isolated signal was not accompanied by corroborating evidence sufficient to rate the domain as serious. Egger's tests were not statistically detectable for BMI, total testosterone, sex hormone-binding globulin, follicle-stimulating hormone, or luteinizing hormone. Formal asymmetry tests were not performed for outcomes with fewer than 10 independent studies.

[5] Indirectness — not serious. Certainty was assessed for the prespecified biochemical and anthropometric outcomes, which were the outcomes addressed by the review question. Ratings therefore apply to these surrogate markers and were not extended to ovarian function, fertility, or symptom outcomes; no outcome was rated down for indirectness on the basis of surrogacy.

Interpretation. Randomized trials started at high certainty. Certainty was downgraded by one level for each serious concern and by two levels for each very serious concern. Negative Hedges' g values indicate lower outcome values with aerobic exercise; positive values indicate higher values. The clinical desirability of direction is outcome-specific.

Certainty categories. High ⨁⨁⨁⨁; Moderate ⨁⨁⨁○; Low ⨁⨁○○; Very low ⨁○○○. Evidence certainty ranged from low to very low: nine outcomes were rated low certainty and four were rated very low certainty.

Abbreviations. AMH, anti-Müllerian hormone; CI, confidence interval; DHEAS, dehydroepiandrosterone sulfate; FSH, follicle-stimulating hormone; GRADE, Grading of Recommendations Assessment, Development and Evaluation; LH, luteinizing hormone; PI, prediction interval; PMOS, polyendocrine metabolic ovarian syndrome; RoB 2, revised Cochrane risk-of-bias tool for randomized trials; SHBG, sex hormone-binding globulin.
